# Aberrant mucin expression and keratinization distinguishing severe from mild asthma revealed by interpretable machine learning

**DOI:** 10.1172/jci.insight.202442

**Published:** 2026-07-30

**Authors:** Sagar L. Kale, Augusta Vincent, Mark A. Ross, Isha Mehta, Michael J. Calderon, Richard P. Ramonell, Himanshu Setya, Jessica C. McCreary-Partyka, Huijuan Yuan, Stephanie A. Christenson, Prescott G. Woodruff, Mario Castro, Kaharu Sumino, Nizar N. Jarjour, Loren C. Denlinger, Benjamin Gaston, Eugene R. Bleecker, Deborah A. Meyers, Wendy C. Moore, Elliot Israel, Bruce D. Levy, David Mauger, Serpil Erzurum, Anthony Newbrough, Taylor J. Nee, Prabir Ray, Claudette M. St. Croix, Sally E. Wenzel, Jishnu Das, Anuradha Ray, Marc C. Gauthier

**Affiliations:** 1 Division of Pulmonary, Allergy, Sleep, and Critical Care Medicine, Department of Medicine, and; 2 Department of Immunology, University of Pittsburgh School of Medicine, Pittsburgh, Pennsylvania, USA.; 3 Department of Cell Biology, University of Pittsburgh, Pittsburgh, Pennsylvania, USA.; 4 University of Pittsburgh School of Medicine, Pittsburgh, Pennsylvania, USA.; 5 Division of Pulmonary, Critical Care, Sleep and Allergy, Department of Medicine and Cardiovascular Research Institute, UCSF, San Francisco, California, USA.; 6 Division of Pulmonary, Critical Care and Sleep Medicine, University of Kansas School of Medicine, Kansas City, Kansas, USA.; 7 Division of Pulmonary and Critical Care Medicine, Washington University in St. Louis, St. Louis, Missouri, USA.; 8 Division of Allergy, Pulmonary and Critical Care Medicine, University of Wisconsin School of Medicine, Madison, Wisconsin, USA.; 9 Riley Hospital for Children and Indiana University School of Medicine, Indianapolis, Indiana, USA.; 10 Division of Genetics, Genomics and Precision Medicine, Department of Medicine, University of Arizona, Tucson, Arizona, USA.; 11 Section on Pulmonary, Critical Care, Allergy & Immunologic Diseases, Wake Forest School of Medicine, Winston-Salem, North Carolina, USA.; 12 Pulmonary and Critical Care Medicine Division, Department of Medicine, Brigham and Women’s Hospital and Harvard Medical School, Boston, Massachusetts, USA.; 13 Division of Statistics and Bioinformatics, Department of Public Health Sciences, Pennsylvania State University, University Park, Pennsylvania, USA.; 14 Lerner Research Institute, Respiratory Institute, Cleveland Clinic, Cleveland, Ohio, USA.; 15 Department of Environmental and Occupational Health, and; 16 Center for Systems Immunology, University of Pittsburgh, Pittsburgh, Pennsylvania, USA.

**Keywords:** Immunology, Pulmonology, Asthma

## Abstract

Type 2 (T2) immune cells dominate the airways of patients with mild-moderate asthma (MMA) with a more complex type 1 (T1)-T2 mixed immune response evident in treatment-refractory severe asthma (SA). We hypothesized that comparing the transcriptomes of the airway epithelium of patients with SA and MMA would reveal molecular signatures associated with more severe disease in the context of a complex immune response. Using our interpretable machine learning tool, SLIDE, meaningful latent factors (context-specific gene co-expression networks) were revealed that distinguished SA from MMA. Unexpectedly, an aberrant high expression of normally host-protective, membrane-tethered, and IFN-inducible mucins, *MUC1* and *MUC4*, was identified in SA. Gene networks in the significant latent factors discriminating SA from MMA corresponded to enrichment of a keratinization program in SA airways. Keratinization was marked by increased expression of the stress keratin KRT16, signifying squamous metaplasia suggesting adaptive reprogramming of the airway epithelium in response to chronic stress. These mucins and KRT16 were inversely associated with lung function in 2 separate asthma cohorts. Imaging of endobronchial biopsies revealed significantly higher KRT16 protein expression in SA compared with MMA that strongly correlated with MUC1 protein expression. Our study identifies dysregulated host-protective and maladaptive repair responses in SA distinguishing from MMA.

## Abstract 2

Advanced machine learning revealed abnormal airway repair responses in severe, but not milder, asthma that are linked to more severe disease.

## Graphical abstract

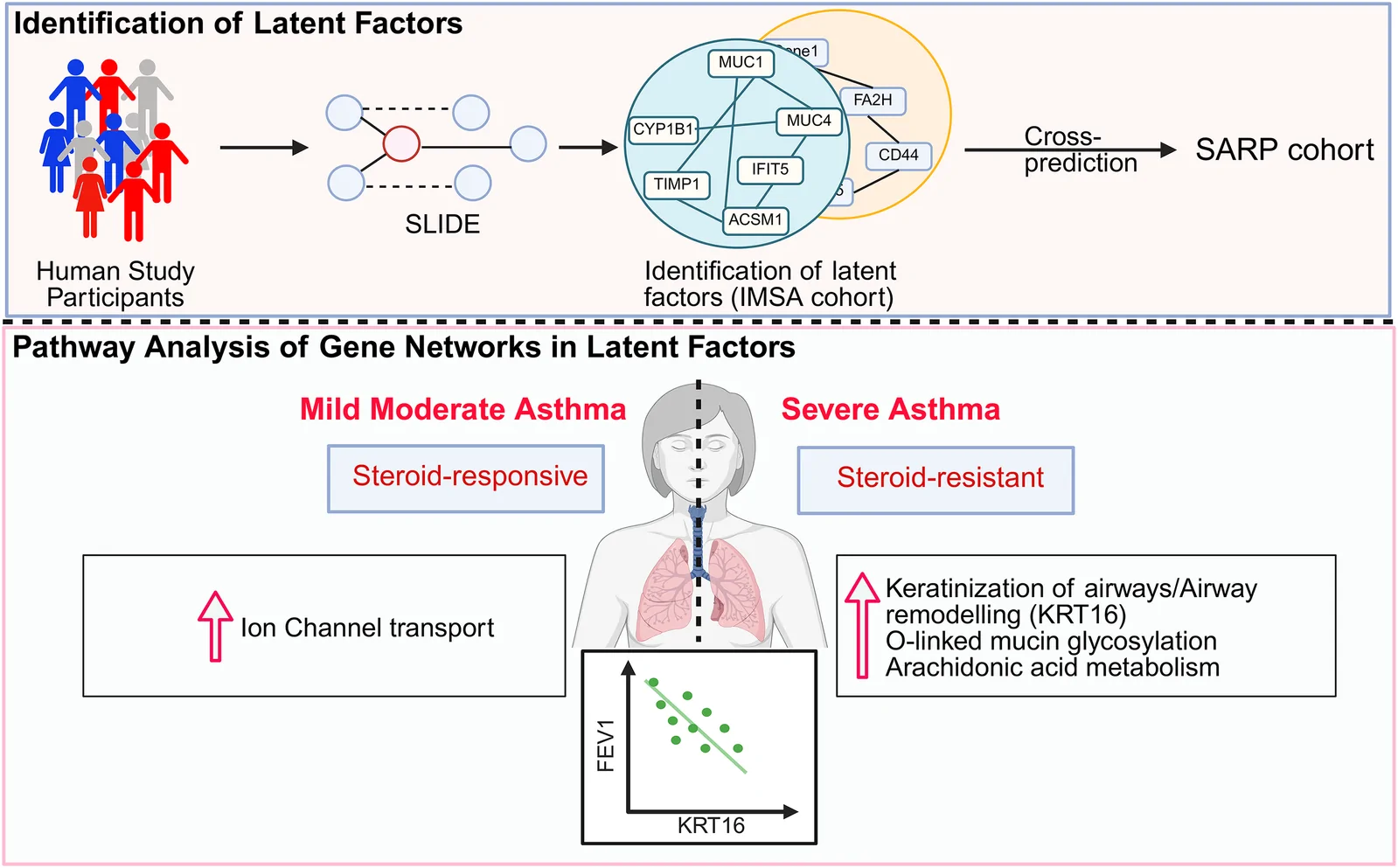

## Introduction

Asthma is a chronic, heterogeneous inflammatory disease of the airways characterized by variable airflow obstruction, bronchial hyperresponsiveness, and airway remodeling (1, 2). While in many patients inhaled corticosteroids (CS) and bronchodilators can achieve optimal disease management, this is not the case in 5%–10% of individuals who are clinically diagnosed as having severe asthma (SA) (3, 4). In these patients, asthma symptoms remain poorly controlled despite high-dose CS therapy, which leads to significant morbidity. Historically, asthma has been viewed as a disease largely driven by a heightened type 2 (T2) immune response associated with increased airway inflammation comprising Th2 cells and additional cell types, such as group 2 innate lymphoid cells that also produce T2 cytokines (5–7), an influx of eosinophils into the airways, an increase in airway hyperreactivity, mucus hypersecretion, and airway remodeling (8). Insights from research in the past decade have replaced this traditional predominantly T2-mediated view of asthma, especially SA, with recognition of a broader, more heterogeneous immune dysregulation that includes type (T1), mixed T1-T2, as well as Th17 immune response in some (8–13). T2-targeted biologic therapies have shown efficacy in SA with many patients achieving significant improvement and even remission of disease (7, 14–16). However, these therapies are not universally effective in SA and many patients with evidence of T2 inflammation show either partial or no response to T2-directed therapies (17). This suggests the presence of additional pathologies in subsets of patients with SA that cannot be controlled by high-dose CS and T2-targeted biologics. Understanding the consequences of complex immune dysregulation, especially its impact on airway epithelial cells, holds promise for improving outcomes in patients with SA.

Immune responses cross-regulate each other (18) and the vast body of literature associating T2 inflammation with asthma invited an early hypothesis that deficiency in T1 inflammation plays a role in promoting asthma. However, murine models showed mixed results, with a Th1 response failing to blunt an opposing Th2 response (19) and a therapeutic trial showing that recombinant IFN-γ was ineffective (20, 21). Analysis of T cells in sputum identified increased expression of both T1 and T2 cytokines in asthma compared with that in healthy controls (22) and bronchoalveolar lavage (BAL) cells revealed an increased IFN-γ response in T cells in SA compared with that in mild-moderate asthma (MMA) (13). Notably, exposure of *Ifng^–/–^* mice to a model of SA resulted in loss of the asthma phenotype, while *Il17ra^–/–^* mice had no improvement compared to wild-type (WT) mice (13). Similarly, a mast cell–dependent role for IFN-γ in asthma pathology was described (23). These observations supported T1 inflammation as a CS-resistant pathway of inflammation in asthma. A T1 immune response can be identified in approximately 30% of individuals with SA (12, 13, 24) and this profile is evident across multiple cohorts, including the Immune Mechanisms in Severe Asthma (IMSA) cohort (12), multiple iterations of the NHLBI Severe Asthma Research Program (SARP) in the United States (13, 24–27), cohorts in Europe including Unbiased Biomarkers for the Prediction of Respiratory Disease Outcomes (UBIOPRED) (11, 28–30), and in pediatric studies (31). Analysis of microarray data generated by the SARP I/II cohort showed that approximately 20% had elevated expression of *IFNG* in the BAL and that this correlated with disease severity, oral CS use, and exacerbations (25). Data from this cohort demonstrated independent elevations in T1 and T2 inflammatory pathways in SA, with the subgroup harboring mixed T1-T2 inflammation and elevated fraction of exhaled nitric oxide (FeNO) displaying the most severe clinical manifestations (27). Similarly, sputum RNA-seq data derived from the SARP III cohort associated a combined T1 and T2 response with the most severe disease and with blunted clinical response to systemic CS treatment (32).

In this study, we investigated whether airway epithelial signatures, analogous to differences observed in immune responses between severe and milder asthma, can demarcate clinical severity. Toward this end, we employed a machine-learning approach to epithelial RNA-seq data generated from our asthma patients enrolled in IMSA (12, 24–26). The transcriptomes of airway brushings obtained from individuals with a clinical diagnosis of SA versus those with MMA and healthy controls (HCs) were analyzed using an unsupervised interpretable machine learning algorithm, named Significant Latent Factor Discovery and Exploration (SLIDE), that can identify latent factors (hidden context-specific groups of features) underlying outcomes of interest, including disease phenotypes (33). This multivariate approach moves beyond a conventional predictive modeling approach to identify not just biomarkers underlying outcomes of interest but actually infer putative mechanisms (34–37). Our analysis revealed unexpected changes in the airway epithelium in SA compared with that in MMA that associated with lung function decline and asthma exacerbations in 2 independent asthma cohorts: IMSA and SARP.

## Results

### Epithelial gene expression to determine potential airway drivers of clinical severity.

Since chronic exposure of the airways to a complex inflammatory milieu in SA has the potential to compromise the airway epithelium and contribute to worsening of lung function, we asked whether gene networks at play in the airways of patients with a clinical diagnosis of SA versus MMA might reveal aberrant steroid-refractory expression of molecules and pathways that distinguish severe from milder disease. To better clarify this question and understand airway-level drivers of asthma severity, we turned to our human IMSA cohort. Of the 41 participants who had BAL cells analyzed, 39 also had paired RNA-seq data from airway brushings, with many of these patients displaying a complex mixed T1-T2 immune response (12, 38).

The participants included HCs (*n* = 7), participants with MMA (*n* = 15), and those with SA (*n* = 17) (Table 1). Groups were generally well balanced, with the exception of a trend toward lower median age in the MMA group (*P* = 0.06) and increasing BMI with asthma severity (*P* = 0.01). SA had lower percentage predicted forced expiratory volume in 1 second (FEV1), as expected, but no significant difference was noted in T2 biomarkers, including absolute blood eosinophil counts (*P* = 0.61) or FeNO (*P* = 0.62). Notably, all participants were on background medications prior to their bronchoscopy, which likely contributed to the lack in variability of T2 biomarkers between groups. In the SA group, 3 participants were on biologic therapy for SA (2 on omalizumab and 1 on reslizumab), while 1 participant was on mycophenolate mofetil for asthma control. IMSA allowed past smokers with a total smoking history of less than 10 pack years and more than 12 months abstinence from cigarettes to participate; past smokers were evenly distributed across the severity groups (*P* > 0.99).

### Use of SLIDE to discriminate MMA from SA identifies key latent factors and associated gene networks.

We sought to elucidate differences in expression programs in the airway epithelium between SA, MMA and HC. Univariate differential expression analyses revealed moderate differences in expression profiles across the 3 groups (Supplemental Figure 1; supplemental material available online with this article; https://doi.org/10.1172/jci.insight.202442DS1), but the identified differentially expressed genes (DEGs) did not correspond to strong coherent changes in specific biological pathways. We hypothesized that this was likely due to these conventional univariate analyses being underpowered at these sample sizes. Furthermore, DEGs are simply individual correlative markers that discriminate between conditions, but do not necessarily reflect coordinated programs or mechanisms. Therefore, we used SLIDE (33), an interpretable machine learning approach recently developed by us, that moves beyond the discovery of biomarkers to the inference of putative causal factors. SLIDE provides a robust framework with rigorous statistical guarantees regarding unique identifiability and inference, incorporates nonlinear relationships, and has rigorous false discovery rate (FDR) control. SLIDE-derived latent factors (LFs) are fundamentally different from LFs derived from unsupervised methods like MOFA^+^ and scVI, which are not anchored on an outcome of interest. Post hoc testing of association of such LFs one at a time does not have overall FDR control (we examine all LFs simultaneously) and also has no guarantees on inference (33). We have successfully used SLIDE to identify mechanisms in a wide range of contexts, including systemic sclerosis and type I diabetes (33), asthma (34), schistosomiasis (35), COPD (36), and morphea (39).

**Figure 1:**
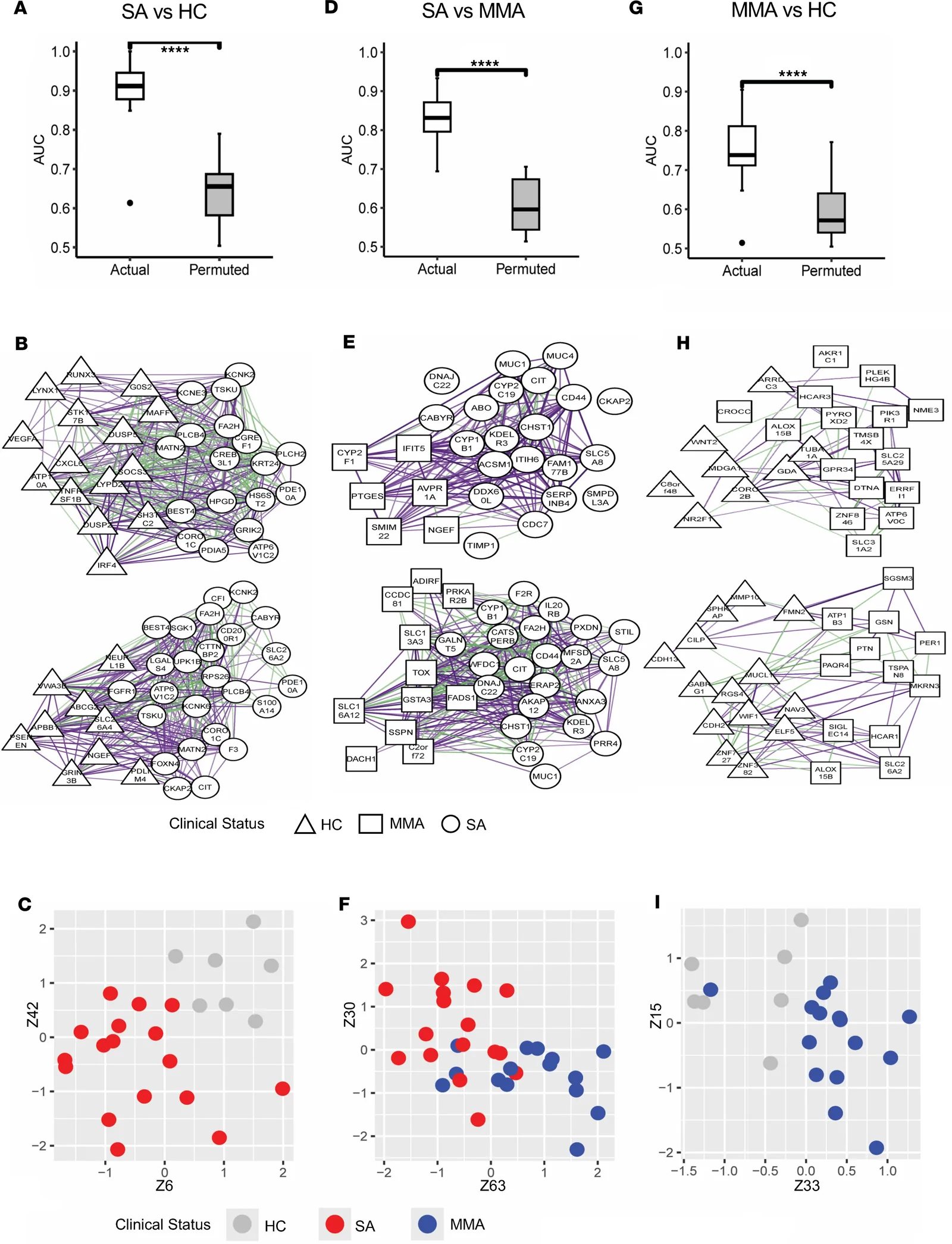
SLIDE analysis of airway brushing RNA-seq data in HCs and participants with MMA or SA. (**A**) Distributions shown correspond to performance of SLIDE models fitted on actual data versus those fitted using permuted labels (negative controls) across replicates of k-fold cross-validation. Bounds of the box represent the first and third quartile, respectively. The line inside the box corresponds to the median. Whiskers correspond to 1.5 times the interquartile range, and dots represent values beyond that. ****P < 0.0001. *P* values calculated from a permutation test. (**B**) Correlation network of features in latent factors (LFs) 6 and 42, which are 2 of 3 latent factors discovered by SLIDE differentiating SA from HC. (**C**) Scatter plot with SA and HC *z* scores, a weighted average of each patient’s transcriptomic expression in terms of the features present within each LF. The *x* and *y* axes represent each participant’s respective LF score. Dots are color coded by participant condition. (**D**–**F**) The corresponding data for SA versus MMA comparison, with correlation network of features in LFs 63 and 30 depicted, which are 2 out of 5 LFs discovered by SLIDE differentiating SA from MMA. (**G**–**I**) For MMA versus HC comparison, correlation network of features in LFs 15 and 33 shown are 2 of 4 LFs discovered by SLIDE differentiating MMA from HC. For correlation networks, triangle nodes (Δ) indicate greater expression in HCs, whereas squares (□) and circles (○) represent the same for patients with MMA and SA, respectively. Purple edges indicate positive correlation, and green edges show negative correlation. Edges are only present if they have a strength of |*R*| > 0.4. Edge thickness indicates strength of correlation. Only nodes that passed a loading and AUC threshold were included.

First, we focused on differences between SA and HC. SLIDE unveiled significant differences (AUC ≈ 0.9 across replicates of *k*-fold cross-validation framework, *P* < 0.01 using permutation testing) in expression programs between SA and HC (Figure 1A). The model included 3 significant LFs that corresponded to context-specific coexpression programs that discriminated between patients with SA and HC (Figure 1B and Supplemental Figure 2A). For deeper biological inference, we further focused on 2 key LFs reflective of higher immune activation, keratinization, and calcium signaling in SA. Just these 2 LFs also provided excellent discrimination between the groups (Figure 1C; each dot corresponds to a patient visualized in LF space). Similar analyses for the SA and MMA groups also revealed significant differences (Figure 1D, AUC ≈ 0.85 across replicates of *k*-fold cross-validation framework, *P* < 0.01 using permutation testing) using 5 significant LFs (Figure 1E and Supplemental Figure 2B). Again, for biological significance, we focused on 2 key LFs reflective of epithelial biology (each including the genes *MUC1* and *MUC4*) that were themselves sufficient to provide excellent discrimination between the groups (Figure 1F). Notably, *MUC1* was present in all 5 significant LFs, while MUC4 was present in 2 of the significant LFs. LFs are by construction overlapping expression programs to reflect pleiotropic functions of genes, and presence of a gene in multiple LFs corresponds to importance in the model. Interestingly, while membrane-tethered *MUC1* and the related mucin *MUC4* are generally host-protective (40–42), they were reflective of a program associated with higher disease severity in this case. We previously reported T2/IL-13–driven sialylation of the N-terminus of MUC4β, an isoform of MUC4, that caused epithelial dysfunction (43). *MUC5AC*, which encodes secreted mucin and is commonly associated with asthma (41, 42), was not in any of the significant LFs. We used rigorous *k*-fold cross-validation, as previously described (33, 35), to demonstrate that the identified LFs were predictive on held-out data.

Thus, the SLIDE model picked up what we believe is a novel expression program not appreciated earlier. Interestingly, the LFs included some genes not identified in the conventional differential gene expression analyses. This is by design, as genes that do not meet traditional univariate significance thresholds can still be part of a core overall multivariate signature (due to additive effects) that is necessary and sufficient to infer differences between the groups of interest. Corresponding analyses for the MMA and HC groups also provided significant differences using 4 significant LFs (Figure 1, G–I, and Supplemental Figure 2C, AUC ≈ 0.75 across replicates of *k*-fold cross-validation framework, *P* < 0.01 using permutation testing). As expected, the most significant difference (AUC ≈ 0.9) was between the SA and HC groups as these reflect two ends of the spectrum. However, unexpectedly, the difference between SA and MMA (AUC ≈ 0.85) was higher than the difference between MMA and HC (AUC ≈ 0.75), suggesting that SA and MMA are more divergent in terms of expression profiles than MMA are from HC (Figure 1, A, D, and G).

**Figure 2:**
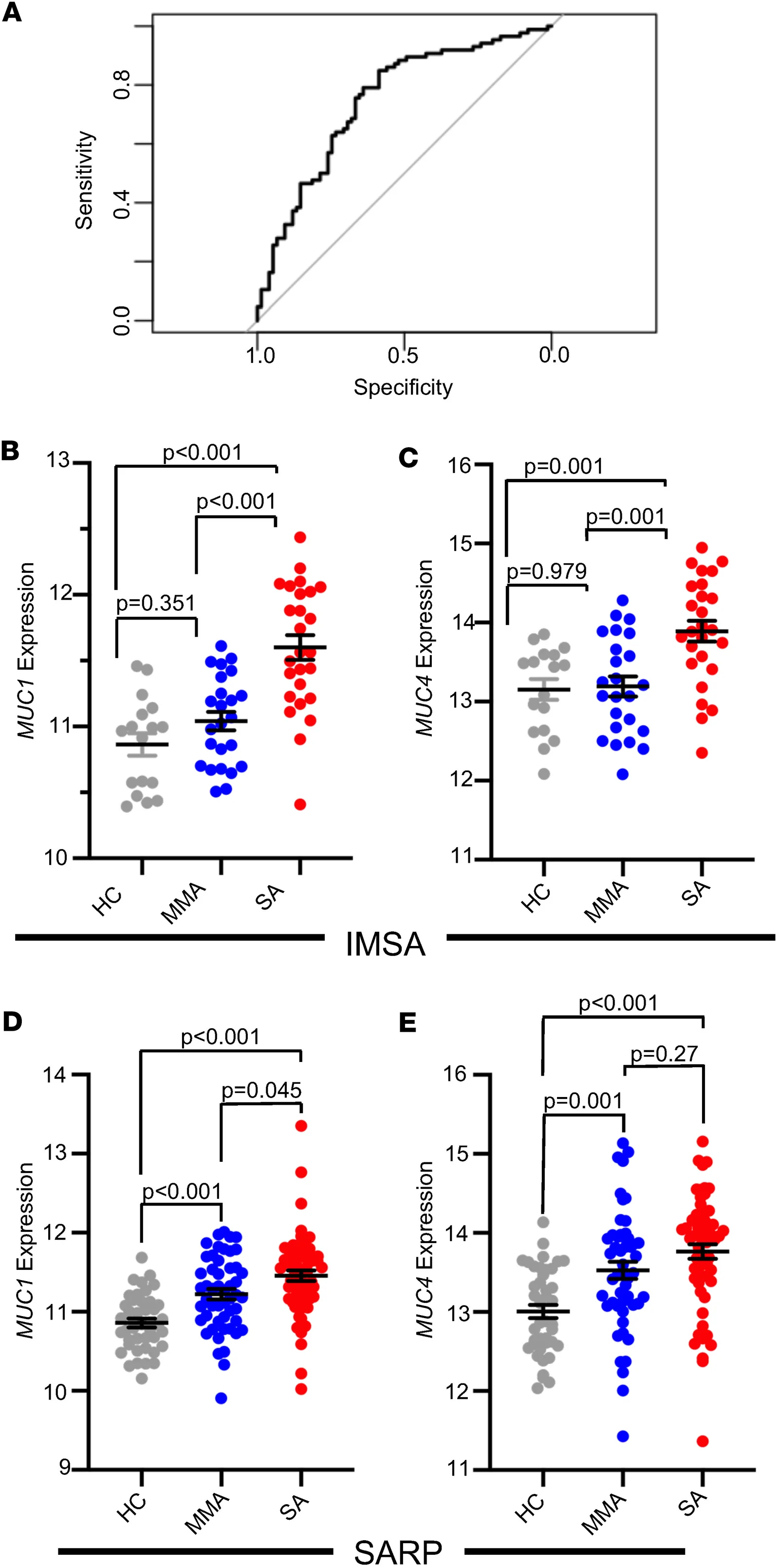
Cross-prediction using the model trained solely on the IMSA cohort to the SARP cohort (model blinded entirely to the SARP cohort for training) and expression of membrane-tethered mucins MUC1 and MUC4 in bronchial epithelial brushings of study participants. (**A**) Five significant latent factors were derived from the SA versus MMA comparison based on the IMSA cohort (using corresponding airway brushing bulk RNA-seq data). This model was used to cross-predict on the SARP cohort (85 SA and 65 MMA) and achieved an AUC of 0.75 (test cohort). (**B**) Analysis of *MUC1* and (**C**) *MUC4* expression in bulk RNA-seq data of bronchial epithelial brushings derived from the IMSA cohort and (**D**) and (**E**) are the corresponding data derived from bronchial brushings of the SARP cohort. Kruskal-Wallis with Dunn’s post hoc testing (**B**–**E**).

We next investigated whether the significant LFs revealed by SLIDE could be validated in an independent cohort. The NHLBI Severe Asthma Research Program (SARP) is a multicenter cohort study of SA that also includes MMA and HCs for comparison. The participants in SARP are deeply characterized clinically and undergo investigative procedures to acquire samples for analysis. A subgroup of 152 individuals (41 HC, 48 MMA and 63 SA; Supplemental Table 1) underwent bronchoscopy with airway brushings for RNA, which were then processed and analyzed for bulk RNA-seq. Indeed, the LFs were cross-predictive in SARP (AUC ≈ 0.75) (Figure 2A). Since the SLIDE model was trained using only the IMSA cohort (includes identification of the composition and relative importance of the significant LFs), and this was evaluated on a completely orthogonal cohort (SARP), this represents a stringent validation on completely held-out data. Thus, 2 levels of evidence — *k*-fold cross validation of our data demonstrating model robustness on held-out data and cross-prediction on an orthogonal cohort — further strengthened the generalizability of the identified LFs.

### Core LF genes MUC1 and MUC4 are increased in SA in both IMSA and a confirmatory cohort (SARP).

We sought to validate these findings in the full IMSA cohort as well as confirm them in a second independent cohort. We examined *MUC1* and *MUC4* expression in bronchial epithelial cells (BECs) recovered by airway brushings from RNA-seq data for the full IMSA cohort (*n* = 68). We observed significant elevations in *MUC1* and *MUC4* expression in SA compared with HC and compared with MMA in this cohort (*P* < 0.001; Figure 2, B and C). These data confirmed that increased expression of these genes identified in the significant SLIDE LFs were associated with disease severity in the cohort at large. To validate our findings, we also investigated *MUC1* and *MUC4* expression in the separate, larger cohort, SARP. Analyzing the dataset for *MUC1* and *MUC4* expression showed a similar pattern to the IMSA cohort, with significant elevations in SA compared with HC (*P* < 0.001; Figure 2, D and E). Notably, the MMA expression differed from IMSA, with increased expression in the MMA group compared with HC (*P* < 0.001). However, SA mean expression remained significantly elevated compared with MMA in the case of *MUC1* (*P* = 0.045). While mean expression of *MUC4* in SA was also higher than in MMA, the difference did not reach statistical significance (*P* = 0.27). This likely reflects the differences in the regional heterogeneity of participants between the IMSA cohort, local to the University of Pittsburgh, and the SARP cohort, which is multi-site cohort. Notably, there is substantial geographic variability in exposures to particulate matter such as PM_2.5_ across North America, with western Pennsylvania exhibiting some of the highest levels of PM_2.5_ and its constituents (44, 45). These environmental differences may contribute to cohort-specific gene expression patterns. Yet, despite these differences in the cohorts, the patterns of *MUC1* and *MUC4* elevation in both suggested association of these mucins with asthma severity.

### MUC1 and MUC4 expression correlate with clinical measures of asthma severity.

**Figure 3:**
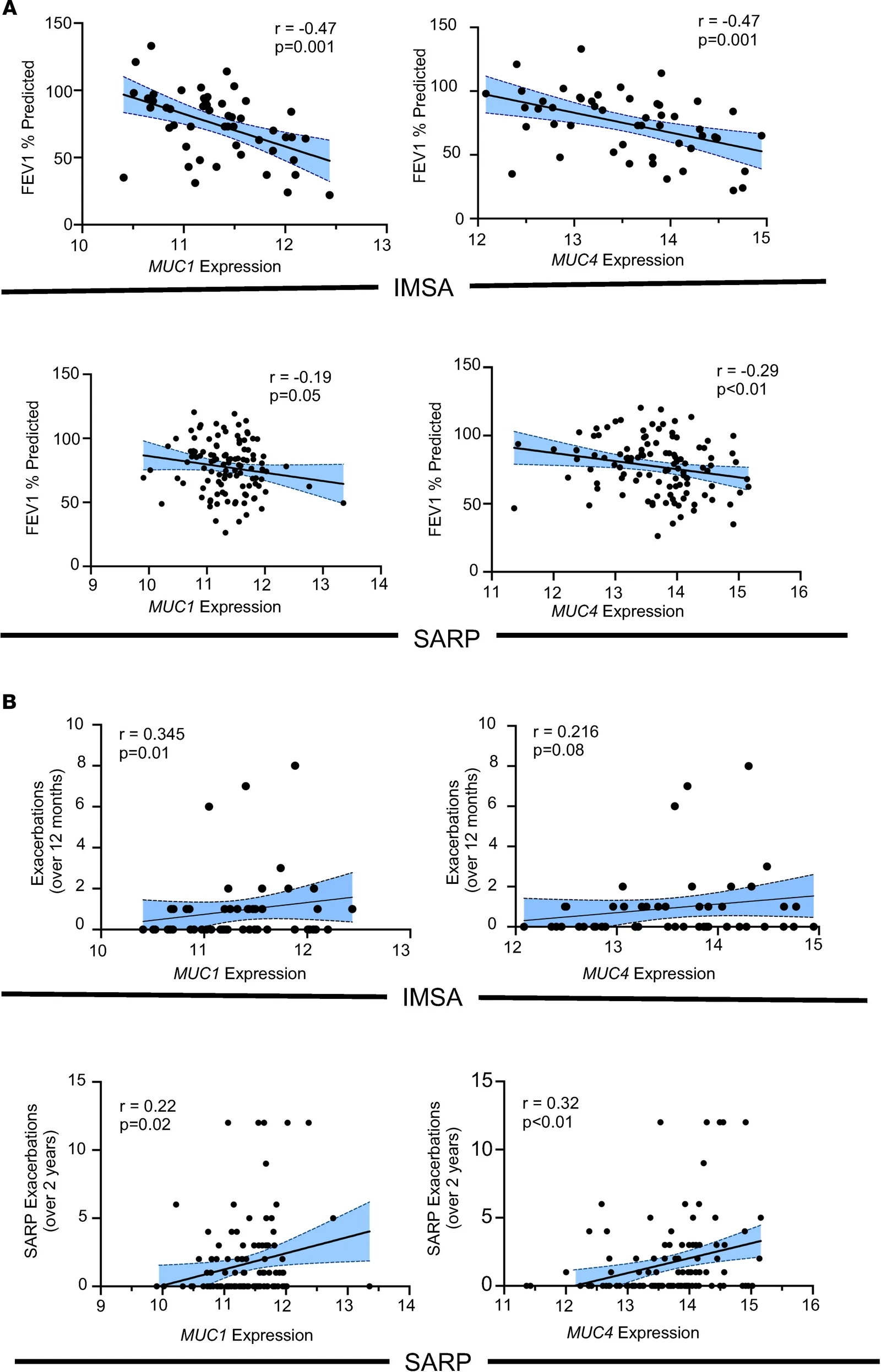
Asthma clinical correlations with *MUC1* and *MUC4* expression. (**A**) FEV1% predicted plotted against expression of *MUC1* and *MUC4* obtained from RNA-seq data of airway brushings from the IMSA and SARP cohorts. (**B**) Number of exacerbations in the 12-month period of cohort enrollment in IMSA was plotted against expression of *MUC1* and *MUC4* obtained from RNA-seq data of airway brushings from the IMSA cohort. In the case of the SARP cohort, the data show number of cumulative prospective exacerbations occurring from baseline visit through year 3 in SARP was plotted against expression of *MUC1* and *MUC4* obtained from RNA-seq data of airway brushings from the SARP cohort. Spearman’s nonparametric correlations, linear regression (solid line) with 95% CI (shaded blue) shown on each scatter plot for reference.

We next assessed the factors identified by SLIDE for correlation with clinical parameters of disease. We examined the correlation of *MUC1* and *MUC4* with percentage predicted FEV1 (race neutral equations). Both genes showed a strong negative correlation with FEV1 in IMSA (*MUC1* *r* = –0.47, *P* < 0.001; *MUC4* *r* = –0.47, *P* < 0.001) (Figure 3A). In the case of the SARP cohort, the correlations for the mucin genes were not as strong but remained significant (*MUC1* *r* = –0.19, *P* = 0.05; *MUC4* *r* = –0.29, *P* < 0.01) (Figure 3A).

While lung function is a major determinant of asthma symptoms and physiological burden, exacerbations are the other primary driver of asthma morbidity. We assessed the correlation of *MUC1* and *MUC4* with prospective exacerbations in the IMSA cohort (individuals reported exacerbations at 12-month visits after bronchoscopy). *MUC1* showed a positive correlation with the number of exacerbations in that 12-month window (*MUC1* *r* = 0.345, *P* = 0.01; Figure 3B). *MUC4* showed a smaller correlation but did not reach significance (*r* = 0.216, *P* = 0.08; Figure 3B). We also reviewed data on exacerbations in the SARP cohort. Here, both genes showed significant associations with prospective exacerbation numbers over years 1–3 in the SARP cohort (*MUC1* *r* = 0.22, *P* = 0.02; *MUC4* *r* = 0.32, *P* < 0.01; Figure 3B). These data show that *MUC1* and *MUC4* associate with both decreased lung function as well as exacerbations, 2 of the primary determinants of clinical asthma severity. However, our data revealed differences in relationships between *MUC1* and *MUC4* expression and disease features between the IMSA and SARP cohorts, raising the possibility that geographically driven environmental factors contribute to these differences.

### Extracellular collagen deposition does not distinguish between SA and MMA.

**Figure 4:**
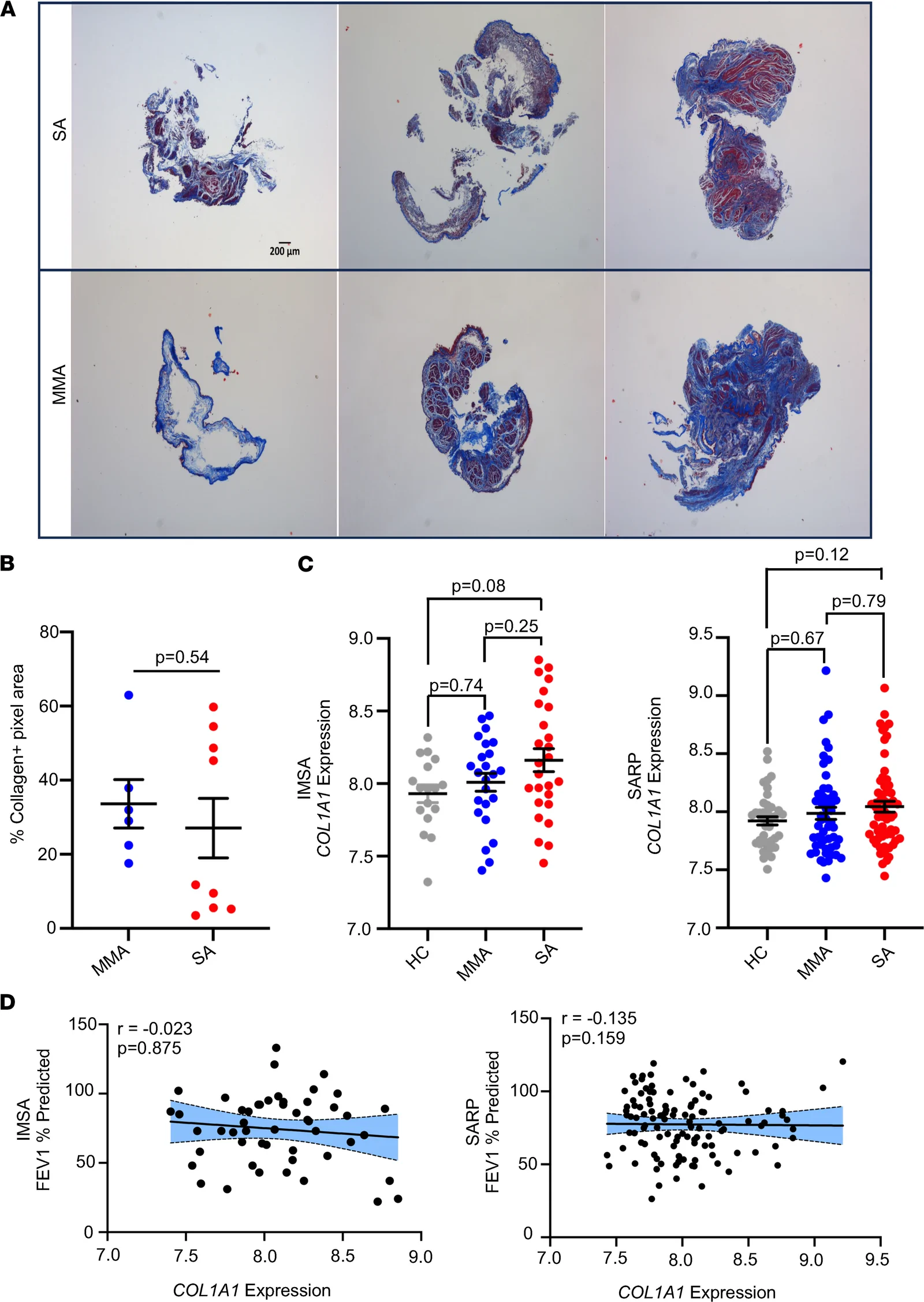
Collagen deposition in endobronchial biopsy specimens from patients with asthma. (**A**) Representative images (magnification, ×4) of Masson’s trichrome–stained endobronchial biopsies obtained from patients with SA and MMA. Scale bar: 200 μm. (**B**) Collagen quantification as percentage trichrome^+^ pixel area per total tissue area in Masson’s trichrome–stained human endobronchial biopsies obtained from patients with SA (*n* = 9) and MMA (*n* = 6). (**C**) *COL1A1* expression in IMSA and SARP cohorts from bulk RNA-seq of bronchial epithelial brushings by asthma severity. (**D**) FEV1% predicted was plotted against expression of *COL1A1* obtained from RNA-seq of airway brushings from IMSA and SARP cohorts. Data plotted as mean ± SEM. Spearman’s nonparametric correlations, linear regression (solid line) with 95% CI (shaded blue) shown on each scatter plot.

Airway remodeling, marked by increased collagen deposition, is a common feature of both an obstructive airway disease such as asthma and a restrictive lung disease such as pulmonary fibrosis (46, 47). However, no significant difference in collagen content was detected between endobronchial biopsy specimens from patients with SA and MMA when stained with Masson’s trichrome (Figure 4A), as quantified by percentage collagen staining (*P* = 0.54; Figure 4B), consistent with previous observations of no difference in collagen deposition in severe versus milder disease (48). Examination of expression of the type I collagen gene, *COL1A1*, in the IMSA and SARP cohorts showed no significant difference in expression between HC, MMA, and SA (Figure 4C). Finally, we also assessed the association of collagen expression (*COL1A1*) in both the SARP and IMSA cohorts with percentage predicted FEV1, which revealed a lack of association (*P* = 0.875 for IMSA, *P* = 0.159 for SARP; Figure 4D).

### CEACAM5 is a DEG for SA compared with MMA and associated with MUC1 and MUC4.

While *CEACAM5* was not included in the identified LFs from the SLIDE model, it was one of the most strongly expressed DEGs in the analysis of SA versus MMA (Supplemental Figure 1) and its increased expression was previously observed in other SA cohorts (28, 49, 50). The absence of *CEACAM5* in the LFs is likely reflective of individual important biomarkers that are not, however, part of an overall gene coexpression program that is necessary and sufficient to distinguish between SA and MMA. Analysis of *CEACAM5* expression in the IMSA cohort at large showed significant elevation in SA compared with both MMA and HC (*P* < 0.01; Supplemental Figure 3A). *CEACAM5* expression also tracked strongly with both *MUC1* (Spearman’s *r* = 0.68, *P* < 0.01) and *MUC4* (Spearman’s *r* = 0.69, *P* < 0.01) (Supplemental Figure 3, B and C). *CEACAM5* also showed negative correlation with percentage predicted FEV1 (*r* = –0.62, *P* < 0.01) and positive correlation with prospective asthma exacerbations (*r* = 0.368, *P* < 0.01) (Supplemental Figure 3, D and E). In the independent SARP cohort, both SA and MMA showed a significantly increased expression of *CEACAM5* compared with HC (*P* < 0.01), although the difference between SA and MMA was not statistically significant (*P* = 0.27; Supplemental Figure 3F). Assessment of *CEACAM5* expression with *MUC1* and *MUC4* expression similarly showed strong correlation (*MUC1* Spearman’s *r* = 0.52, *P* < 0.01; *MUC4* Spearman’s *r* = 0.62, *P* < 0.01; Supplemental Figure 3, G and H). *CEACAM5* showed negative correlation with percentage predicted FEV1 (*r* = –0.39, *P* < 0.01) and positive correlation with prospective asthma exacerbations (*r* = 0.38, *P* < 0.01) (Supplemental Figure 3, I and J).

### Pathway analysis of gene networks identified by SLIDE identify keratinization, airway remodeling, O-linked mucin glycosylation, and arachidonic acid metabolism as discriminating pathways between SA and MMA.

**Figure 5:**
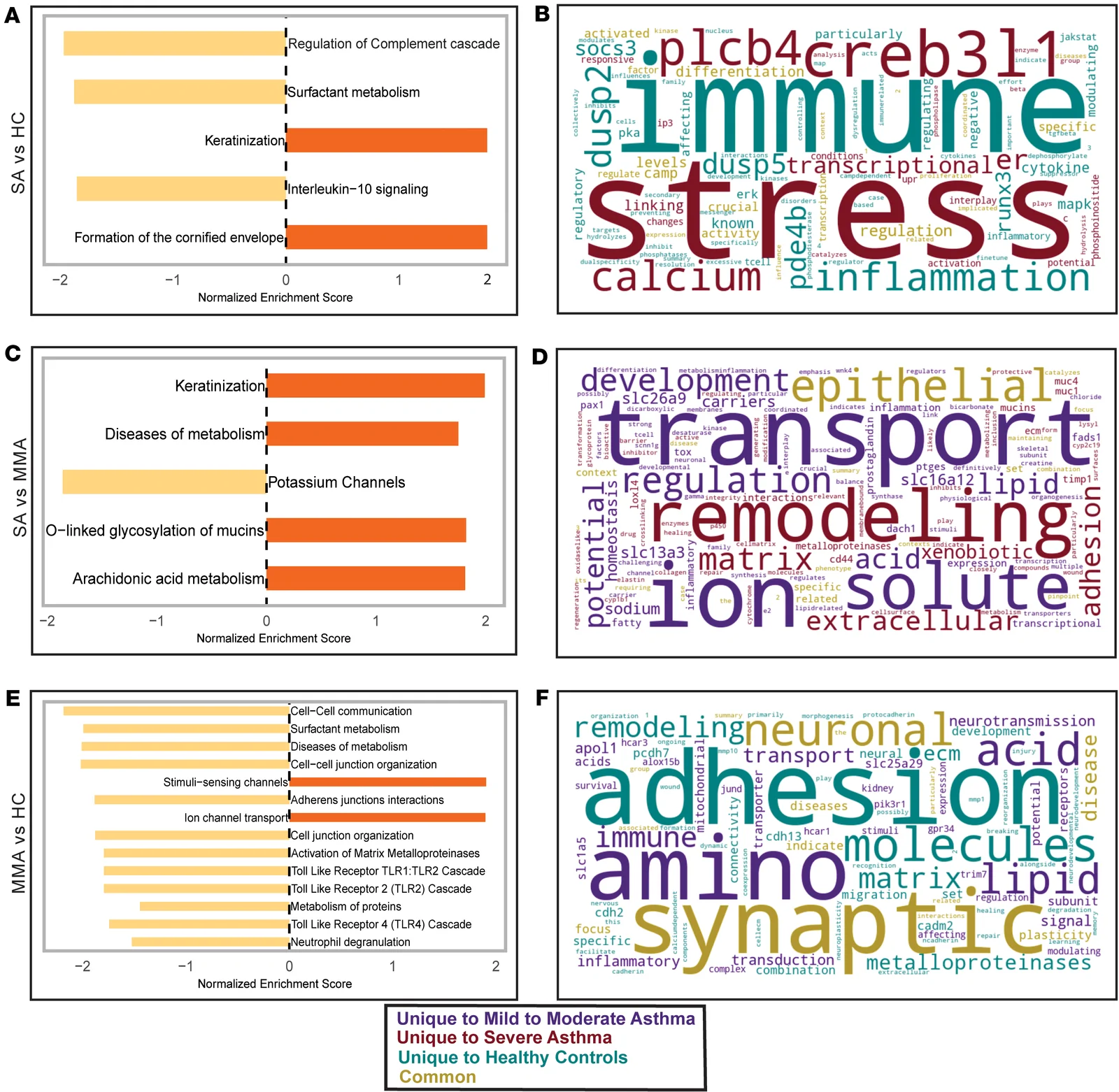
GSEA and GSAI analysis of SLIDE data. (**A**, **C**, and **E**) GSEA plot with pathway on *y* axis and normalized enrichment score (NES) on *x* axis arranged by *q* value. SLIDE features in the (**A**) SA versus HC, (**C**) SA versus MMA, and (**E**) MMA versus HC latent factors (LFs) were input into GSEA to find pathways enriched within the LFs. Pathways with a negative NES are enriched in the participants with lower clinical severity within a given pairwise comparison, and vice versa for positive NES. All pathways significant at *q* < 0.2. (**B**, **D**, and **F**) Word cloud featuring GSAI output interpretation when features present in SLIDE LFs within the (**B**) SA versus HC, (**D**) SA versus MMA, and (**F**) MMA versus HC comparisons were used as input. Burgundy, indigo, and teal words represent terms enriched in SA, MMA, and HC, respectively. Gold words are common between both conditions in each figure.

Next, we focused on elucidating pathways to which these LFs corresponded. We used a 2-pronged approach involving conventional gene set enrichment analysis (GSEA) and a large language model–based (LLM-based) approach — gene set artificial intelligence (GSAI). GSEA revealed enrichment in keratinization in SA when compared with HC or MMA as well as increase in O-glycosylation of mucins (Figure 5, A and C), the latter aligning with the finding of *MUC1* and *MUC4* as core LF genes in SLIDE analysis of SA versus MMA (Figure 1 and Supplemental Figure 2). In addition, compared with MMA, an increase in arachidonic acid metabolism, particularly in relation to 15 lipoxygenase, was also identified in SA (Figure 5C) that we previously described (51, 52), and is associated with ferroptotic processes in airway epithelial cells (53). While GSEA allows us to aggregate small-additive effects of expression differences in the identified genes in coherent pathways, they are inherently constrained by the structure of known pathways. Since LFs reveal novel, context-specific groups of co-expressed genes, they may inform novel biological outcomes not included in established gene sets/pathways used in conventional overrepresentation or gene-set enrichment analyses. To address this, we adopted GSAI (54) to allow unbiased queries of the genes in the LFs against the known literature. GSAI uses a pretrained bidirectional transformer (BERT) model to aggregate LLM responses and comes up with coherent biological process descriptions for these LFs. GSAI has the capacity to uncover novel functional groupings and combinatorial processes that enrichment tests structurally cannot recover since over representation analysis (ORA) or GSEA can only rediscover categories that are already defined. Furthermore, GSAI demonstrates performance equivalent to or better than conventional ORA/GSEA methods in terms of FDR control. The word cloud synthesizes GSAI outputs as term frequencies to highlight key pathway/functional components identified in the LFs. Use of GSAI revealed that while the differences revealed between SA and HC were primarily centered around immune activation and stress responses (Figure 5B), epithelial and matrix remodeling reflecting keratinization as well as altered ion transport were the key differences between SA and MMA (Figure 5, C and D). Although distinct approaches underlie GSEA and GSAI, it was remarkable that both distinguished SA from MMA by signatures of increased airway remodeling while enhanced ion transport was a key feature of MMA. There were more diverse differences between MMA and HC (Figure 5, E and F).

### Keratinization is noted in endobronchial biopsies in SA.

**Figure 6:**
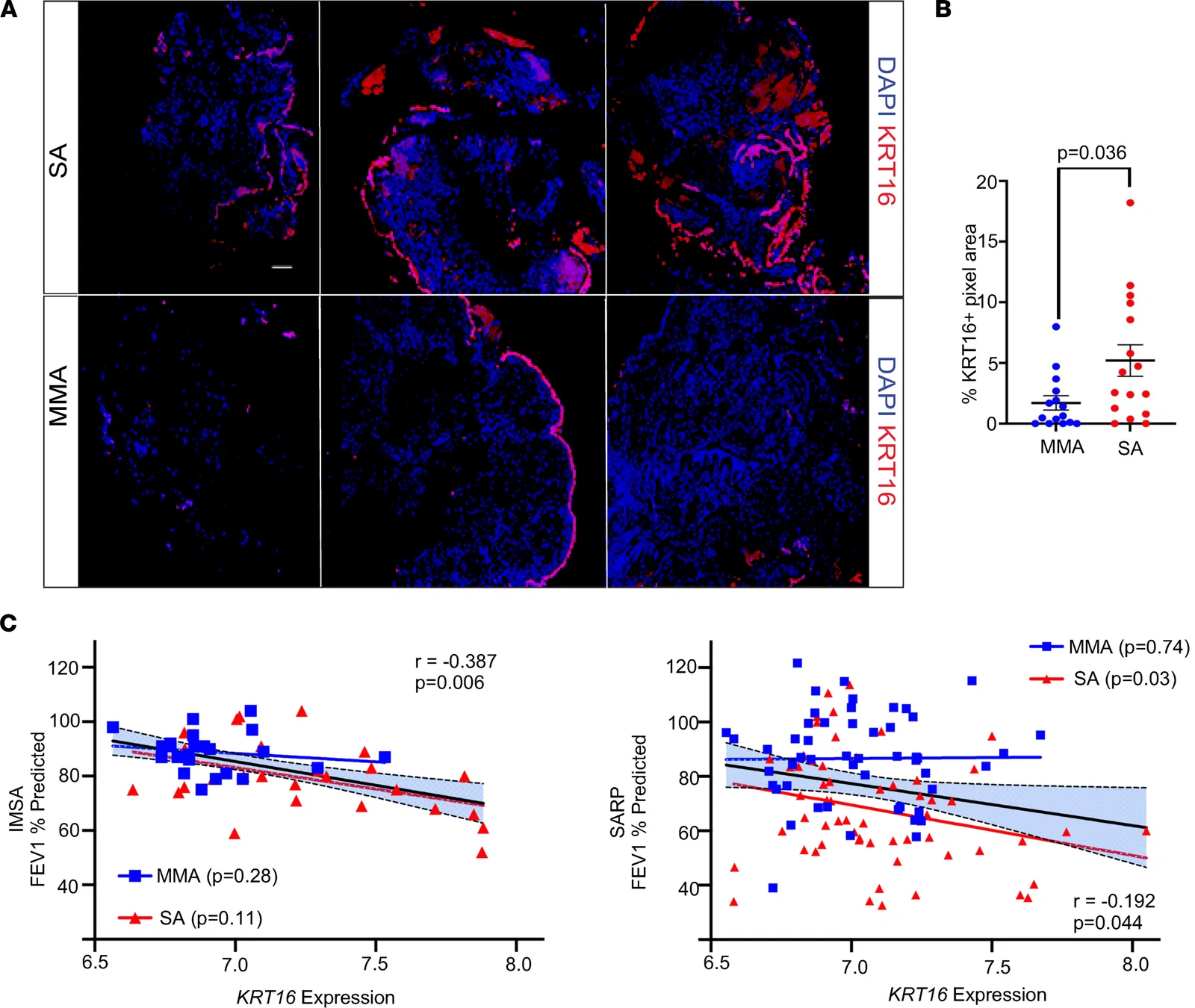
KRT16 protein expression in patients with asthma in the airways of IMSA and relationship between KRT16 expression and lung function in both cohorts. (**A**) Representative immunofluorescence images of KRT16 protein expression in endobronchial biopsies of patients with SA and MMA in IMSA and (**B**) quantification as percentage KRT1^+^ pixel area per total tissue pixel area in the specimens. Data plotted as mean ± SEM and were analyzed by Mann-Whitney *U* test. Scale bar: 200 μm. (**C**) FEV1% predicted plotted against expression of *KRT16* in RNA-seq data of airway brushings from patients with SA or MMA in the IMSA and SARP cohorts. Spearman’s nonparametric correlation with linear regression for all asthma (black with 95% CI) and severity specific linear regression lines (blue in MMA, red in SA) shown.

To further investigate the airway remodeling findings, we examined keratin protein content in endobronchial biopsies from the IMSA cohort. While numerous keratins play a role in keratin formation and deposition, we focused on KRT16, a type I keratin, a common keratin (KRT) gene identified in the GSEA analysis of SA versus MMA as well as SA versus HC LFs, as assessed by SLIDE (Supplemental Table 2). It is important to also note that KRT16 is regarded as a stress keratin and is associated with epithelial injury and diseased epithelia (55, 56). Our analysis identified a statistically significant difference in KRT16 protein levels between MMA and SA (*P* = 0.036) (Figure 6, A and B). To assess clinical outcomes associated with KRT16, we utilized RNA-seq expression data that were available from the full IMSA cohort and in SARP. KRT16 expression showed a significant inverse association with predicted percentage FEV1 in all asthma in both the IMSA (*P* = 0.006) and SARP (*P* = 0.044) cohorts (Figure 6C). Notably, this negative correlation with FEV1 in the SARP cohort was driven exclusively by a negative correlation in SA (*P* = 0.03) with no significant correlation identified in MMA (*P* = 0.74). While a similar pattern was seen in the IMSA cohort, this correlation was not statistically significant likely due to the smaller number of participants (Figure 6C). Collectively, our data suggest that increased keratinization seen in the airways in SA is independent of lung tissue fibrosis, representing a different pathology and correlated with worsening airway obstruction.

### Analysis of effect sizes of genes within the significant SLIDE LFs as a predictor of severity shows unique contributions of MUC1 and MUC4 in a pattern different from traditional T2 asthma–associated genes.

**Figure 7:**
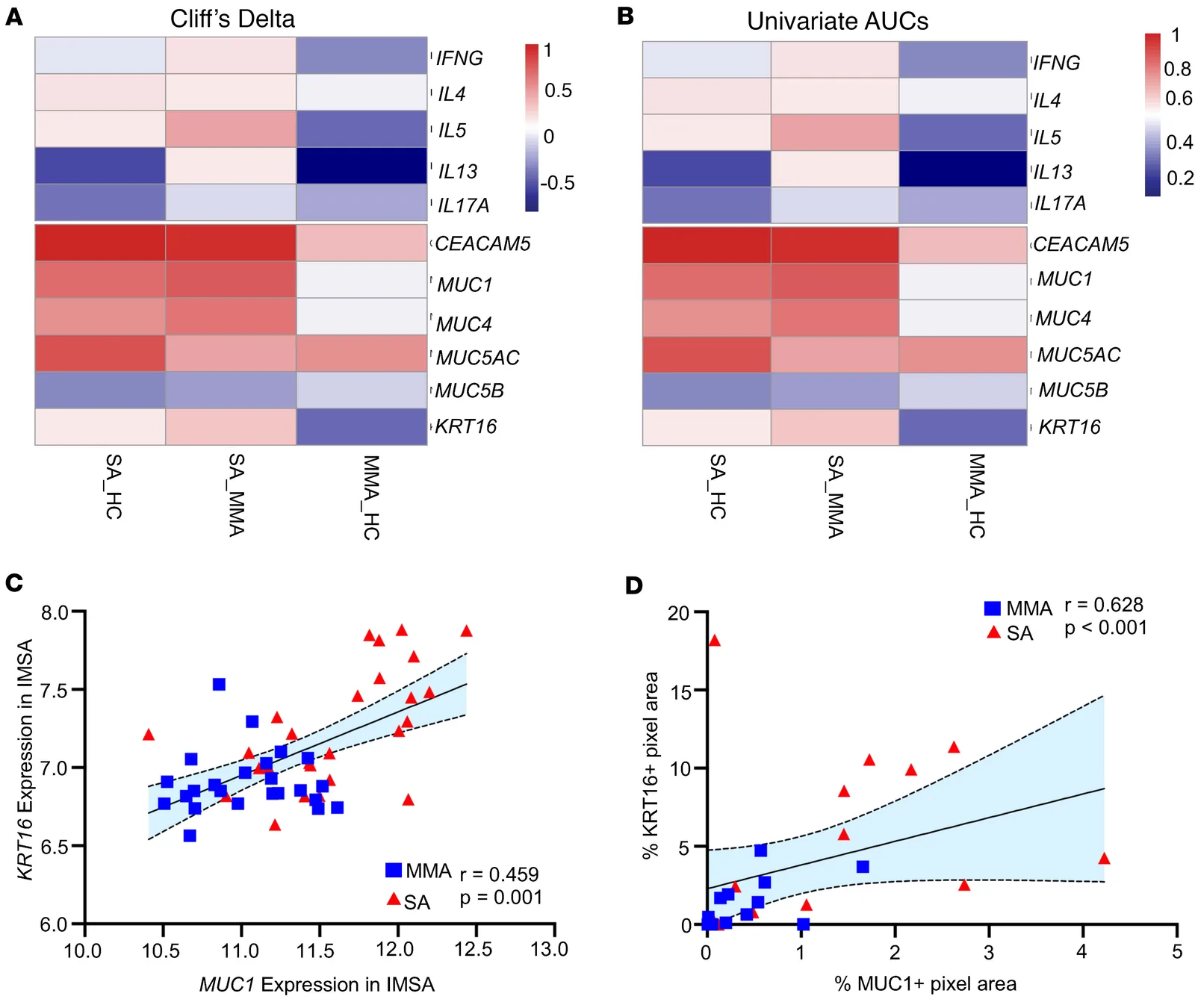
Single genes of interest with discriminatory power across clinical severity. (**A**) Heatmap based on Cliff’s delta values for several genes of interest for the indicated pairwise comparisons. Each row is a specific gene. Blue cells indicate higher expression in the lower severity, and vice versa for red. Cells that are white have no difference between the conditions. (**B**) Heatmap based on univariate AUC values using the same genes. (**C**) Scatter plot of *KRT16* and *MUC1* expression in bronchial brushing RNA-seq data in IMSA (blue = mild to moderate asthma, red = severe asthma). (**D**) Scatter plot of KRT16 and MUC1 protein staining (quantified as the percentage of pixel area of the biopsy positive for staining) in endobronchial biopsies from IMSA participants. Spearman’s correlation for **C** and **D** with linear regression and 95% CIs shown.

We also examined the effect sizes, quantified by Cliff’s delta (Figure 7A) and AUC (Figure 7B), of specific genes (T1, T2, T17, mucins and KRT16) in each of the 3 comparisons of interest. The data first showed that conventional T2 genes *IL4*, *IL5*, *IL13*, *IFNG*, or *IL17A* were not discriminative of MMA from HC. The expression of these genes was actually higher in HC, likely reflective of CS-mediated suppression of cytokine production in MMA (13). In comparisons between SA and MMA, T1 and T2 cytokine gene signatures were higher in SA compared with MMA, illustrating poor CS response in severe disease (2, 13) and presence of T1-T2 interaction in SA (10, 32). However, the most discriminative genes between SA and MMA were the tethered mucins *MUC1* and *MUC4* and the cell adhesion molecule *CEACAM5*, further confirming the importance of these molecules relative to genes we conventionally focus on and target in CS-refractory SA. Consistent with the pathway analysis data, *KRT16* was also a strong predictor of SA versus MMA but did not discriminate between MMA and HC. Since MUC4 and CEACAM5 did not consistently separate SA from MMA in SARP, we focused on MUC1 and KRT16 and examined the relationship between these molecules at both the RNA and protein levels, which revealed a significant correlation (Figure 7, C and D, Supplemental Figure 4).

While active smokers were excluded from the SARP and IMSA cohorts, participants with a prior smoking history of less than 10 pack years and more than 12 months of smoking cessation were allowed to enroll. To confirm that our findings were not related to past cigarette use, we examined *MUC1*, *MUC4*, and *KRT16* expression by prior cigarette use and found no significant difference in expression levels by smoking status in either cohort (Supplemental Figure 5).

## Discussion

Given the profound impact of immune responses on the airway epithelium, we sought to determine whether applying a novel, unsupervised interpretable machine learning tool, SLIDE, designed to identify LFs of disease (33), could uncover signatures in the airway transcriptome associated with distinct clinical presentations of asthma that may not be revealed by univariate differential gene expression analysis. Indeed, SLIDE identified meaningful LFs (context-specific gene coexpression programs) that discriminated between disease states (SA, MMA, and HC) where conventional approaches failed. Unexpectedly, comparison of SA and MMA revealed enrichment of genes encoding the membrane-tethered mucins *MUC1* and *MUC4* across multiple LFs, with *MUC1* consistently represented in every significant LF. By showing association with key features of asthma, such as lung function and asthma exacerbations, in 2 independent asthma cohorts, IMSA and SARP, we show the biological significance of the presence of *MUC1* and *MUC4* in multiple LFs discriminating mild and severe asthma. Pathway analysis using 2 independent approaches to understand the biological significance of the gene networks revealed enrichment of keratinization, O-glycosylation of mucins, and airway remodeling in SA versus MMA. By confirming increased protein expression of KRT16, included in the GSEA gene sets for both SA versus MMA and SA versus HC comparisons, we demonstrate that the airway in SA patients expresses stress keratins that, to the best of our knowledge, have never been heretofore identified in the airways. Stress keratins, such as KRT16, are induced in response to environmental stressors in the context of chronic inflammatory disorders such as psoriasis, in hyperproliferative wound healing epithelium, and squamous cell carcinoma (57–60). In conventional differential gene expression analysis, *CEACAM5* showed significantly higher expression in SA versus MMA, *CEACAM5* being the only differentially expressed transcript that was associated with frequent asthma exacerbations in a study of the U-BIOPRED cohort (28). The association of CEA-related cell adhesion molecules such as CEACAM5, the mucins MUC1 and MUC4, and KRT16 with SA suggests a dysregulated host-protective response as a key underlying aberration in severe disease.

While membrane-tethered MUC1, and the related mucin, MUC4, are generally host-protective when expressed at a low basal level (40–42), in our study, they are reflective of a program associated with higher disease severity. MUC1 has antiinflammatory functions in the respiratory tract that defend against infections by both viral and bacterial pathogens (42, 61–64), while MUC4 has the ability to block penetration of the mucus gel layer into the tissue (40, 41). The membrane-tethered MUC4 subunit MUC4β has the ability to signal via ErbB2 and ErbB3, which in scratch assays has been shown to be an important component of epithelial wound healing (65). In a similar vein, CEACAM5 helps maintain intercellular adhesion forces that are normally in place in healthy airways (66). Existing literature suggests that the levels of mucin expression and O-glycosylation are regulated by the extent of pathogen invasion (64) and pathway analysis showing greater O-glycosylation of mucins in SA compared with MMA suggests higher pathogen burden in severe disease. Indeed, viral transcripts (32) and multiple bacterial species have been appreciated in the airways of patients with SA (67), suggesting that prolonged subclinical infection or disrupted airway microbiome plays a role in epithelial damage triggering a maladaptive repair response.

Increased *MUC1* expression and its excessive O-glycosylation leads to shedding of the N-terminus of MUC1 protein, as described previously in response to cigarette smoke (68). This frees up its C-terminus and triggers activation of the EGF receptor, which has been linked to KRT16 upregulation (69). Since smoking was an exclusion criterion in our asthma cohorts, it will be interesting to determine the nature of insult(s) that results in similar signatures of increased expression of membrane-tethered mucins and increased O-glycosylation in asthmatic airways. Although our study was not able to demonstrate causality between environmental exposures or aberrant immune activation, it is worth noting that *MUC1* and *CEACAM5* are inducible by IFN and STAT1 (70–73) and *MUC4* expression has been reported downstream of T2 signaling (43, 74), providing a convergence of T1-T2 interaction. An IFN-activated STAT1–glucocorticoid receptor cooperation may also underlie high *MUC1* expression in the asthmatic epithelium, similar to findings with CXCL10 expression induced by the same axis, as we previously showed (24). In addition to IFN signaling, cytotoxic functions of both CD4^+^ T cells (11) and CD8^+^ T cells (12, 75), identified in the airways of SA patients, would be expected to promote epithelial damage. MUC4 is structurally different from MUC1 and although its cytoplasmic tail is too short to signal, its cleaved form, MUC4β, may also affect cell adhesion (76). We previously reported that IL-13 causes MUC4β to undergo ST6GAL1-mediated N-sialylation, which inhibited epithelial cell proliferation but promoted goblet cell differentiation, highlighting a pathogenic form of the truncated MUC4 isoform (43).

Barrier disruption and aberrant MUC1 and MUC4 expression and function would inevitably trigger repair programs in the epithelium, which should be finely regulated. However, enrichment of keratinization and airway remodeling pathways in SA suggests a failed attempt to repair, since a healthy airway epithelium should never express keratins that are normally expressed by the skin, hair, and nails to resist mechanical stress. Among the keratin family members, identification of *KRT16* in the keratinization pathway is also strongly suggestive of squamous metaplasia, this keratin being one key marker of squamous cells (77) and also of disease-associated epithelium (56–60). Squamous epithelium is structurally more rigid, keratinized, and less adaptable to mechanical stretch. A recent transcriptomic study of the nasal airway epithelium of children to identify gene networks associated with asthma exacerbations also suggested differentiation of the epithelium toward keratinized squamous cells that associated with asthma exacerbations in the absence of virus infections (78). Airway hillock cells were described as precursors to squamous metaplasia (79). Whether hillock cell differentiation is involved in aberrant repair of the asthmatic airway resulting in keratinization remains to be determined.

While, other than cigarette smoke, particulate matter exposure (44, 45, 80, 81) and pathogen infections (32, 67) would impact the airway epithelium of patients with SA, the role of autoimmune disease in some (82), which can also induce high IFN responses, cannot be ruled out either. Genetic variations in the mucins and KRT16 may also explain how specific protein interactions are impacted by these variants (83), further exacerbating disease risk. Although chronic obstructive pulmonary disease (COPD), primarily caused by cigarette smoking, has a distinct clinical diagnosis, overlap between asthma and COPD is well recognized but the cause has remained an enigma (84). It is tempting to speculate that the common outcome of different forms of chronic epithelial insults — overexpression of molecules such as the membrane-tethered mucins and CEACAM5 that may drive squamous metaplasia marked by aberrant expression of keratins — contribute to the asthma-COPD overlap syndrome and lack of CS-responsiveness in SA (84). Thus, the ultimate end result of keratinization in SA can be interpreted as an attempt to protect barrier integrity in the face of repeated or severe insults. However, this response becomes pathological, ultimately impairing airway function and frequently showing poor response to CS and limited improvement with T2-directed biologics.

The study has some limitations. While our study revealed discriminating LFs comprising unconventional mucin genes that associated with asthma outcome measures, we did not target these mucins in vivo using mouse models of disease to demonstrate causality between increased MUC1 and MUC4 production and decline in large airway function. Airway remodeling with keratinization likely develops gradually in the chronic disease setting of asthma and therefore induction of squamous metaplasia and associated keratinization would require development of new chronic mouse models that would recapitulate the structural changes observed in human disease.

The cross-sectional nature of the study precludes the ability to determine the persistence of these signals over time; however, the correlation of increased MUC1 and MUC4 expression with prospective exacerbation risk would argue in favor of persistent changes over time. Additionally, both IMSA and SARP cohorts recruited participants who were maintained on background therapy, including inhaled CS and some biologic therapies (although the timing of recruitment in SARP III predates the FDA approval of most T2-targeted biologics). While it is possible that some of the signal could be related to steroid effects, it should be noted that traditionally MUC1 is associated with CS sensitivity, while these participants with SA continued to have severe disease despite CS therapy. Furthermore, studying participants on background therapy is more representative of the real-world pathobiology, which may be positively or sometimes adversely impacted by current therapies. As such, the identification of keratinization and abnormal mucin signatures in these patients collectively represents an important finding that may provide novel mechanisms for future therapeutics that can either augment or supplant current broadly targeted options in the correct patient population.

## Methods

### Sex as a biological variable.

We utilized 2 independent cohorts, the Pittsburgh-based IMSA cohort and the multicenter SARP cohort. Both males and females were recruited, with resulting sex distributions reflective of asthma prevalence rates and local demographics. Male and female distributions were similar in both cohorts.

### IMSA cohort.

The IMSA cohort was recruited at the University of Pittsburgh and has been previously described (12). All participants were 18 years old and nonsmokers; participants could be included with past smoking history of less than 10 total pack years smoking history and more than 12 months abstinence from smoking. Participants underwent detailed clinical characterization including detailed asthma history, spirometry, FeNO measurement, and blood collection for complete blood counts including blood eosinophil counts, as previously described (12). Participants underwent research bronchoscopy with bronchial brushings obtained for bulk RNA-seq and endobronchial biopsies obtained via forceps from the contralateral side from the bronchial brushings, as previously described (12). Bulk RNA-seq data were previously reported and are available online in the NCBI Gene Expression Omnibus database (GEO GSE158752) (38).

### SARP cohort.

Previously obtained data from the SARP III cohort were used. Nonsmoking participants between the ages of 18–60 from racially/ethnically diverse backgrounds were recruited at multiple clinical sites. All participants provided informed consent in accordance with local IRBs and data was managed by the Data Coordinating Center at Pennsylvania State University (Penn State). Asthma was determined by physician report and confirmed by the presence of reversible airway obstruction on spirometry or positive bronchoprovocation challenge. Participants meeting the ATS/ERS 2013 definition of severe asthma were classified as severe asthma; the remaining participants were classified as nonsevere or MMA. Participants underwent extensive evaluation, including spirometry, FeNO, asthma clinical assessments, and sputum induction. A subset of participants underwent bronchoscopy with epithelial brushings, which were processed for bulk RNA-seq (85).

### Tissue preparation of endobronchial biopsy specimens and immunofluorescent staining.

Endobronchial biopsies were obtained during bronchoscopy from patients in the IMSA cohort. The biopsy specimens were formalin-fixed and embedded in paraffin (FFPE). The FFPE sections were cut at 5 μm thickness using a rotary microtome and mounted onto glass slides. A set of these sections was used for Masson’s trichrome staining done at Pitt Biospecimen Core at the University of Pittsburgh. Collagen quantification was done using NIS Elements 6.10.01 (Nikon) using the GA3 toolset. The Homologous Area Detection tool was used with the RGB image to segment out the total tissue area. All other segmentations were limited to objects found within the total tissue area. A ratio image of the blue to red pixel intensities was created and the area of positive collagen signal was segmented using standard thresholding. Collagen-positive areas were normalized to total tissue area. The other set of slides was heated overnight on a heating block (60°C) and deparaffinized with xylene. After rehydrating the tissue sections with alcohol/water graded washes and used for immunofluorescent staining. Antigen retrieval was performed in citrate buffer at 90°C and sections were blocked with 5% normal donkey serum in PBS for 45 minutes, followed by washing with PBS containing 0.5% BSA (PBB). The sections were incubated overnight at 4°C with primary antibodies (diluted 1:100) anti-MUC1 (Thermo Fisher, Scientific, MA5-11202, clone MH1 [CT2]) and anti-CD8α (Abcam, ab101-500, clone SP16). Sections were washed 5 times with PBB and incubated for 1 hour in goat anti-rabbit–CY5 (Jackson ImmunoResearch, 111605003) and goat anti–Armenian hamster–Cy3 (Jackson ImmunoResearch, 127165160). Samples were washed 3 times with PBB and a single wash of PBS followed by a 1-minute incubation in Hoechst nuclear stain. Another set was stained with primary antibodies rabbit anti-KRT16 (Abcam, 76416, clone EP1615Y) diluted 1:100 (PBB) and secondary antibody goat anti-rabbit–CY5 (Jackson ImmunoResearch, 111605003). Sections were washed 3 times with PBS and mounted using Gelvatol (23 g polyvinyl alcohol 2000, 50 mL glycerol, 0.1% sodium azide to 100 mL PBS). Imaging and quantification were performed using a Nikon A1 confocal and NIS Elements Software.

### Immunofluorescence image acquisition and analysis.

Immunofluorescence confocal images of ×40 large area scans were analyzed in NIS Elements 6.10.01 using the GA3 toolset. The Homologous Area Detection tool was used with all channels to segment out the total tissue area. All other segmentations were limited to objects found within the total tissue area. Markers were segmented out using standard thresholding to generate binary masks identifying positive signal. Total object area for each binary mask (MUC1 and KRT16) was calculated and final data were normalized to total tissue area.

### Differential expression analysis.

Three patient groups were identified a priori in terms of severity of asthma. These groups were HCs (*n* = 7), patients with MMA (*n* = 15), and patients with SA (*n* = 17). The raw counts received were subjected to pairwise differential expression analysis after filtering, normalization, and variance stabilization using DESeq2 (75).

### Differential expression analysis results.

Each comparison had 21,870 non-zero count features. The SA-MMA comparison and the SA-HC comparison are of particular interest to the goals of this project. For the former, 1,441 genes were identified as significantly different between the 2 conditions at an α level of 0.05. For the SA-HC comparison, slightly fewer genes were discovered to be significant at the same α level, to the tune of 1,087 differentially expressed features. Finally, the MMA-HC comparison resulted in only 5 differentially expressed genes, many of which were driven by outliers among the patients.

### Multivariate machine learning model.

SLIDE, an interpretable machine learning approach, was used to discover significant LFs encompassing transcriptomic differences between the patient groups (33). Asthma severity (SA, MMA, HC) was the outcome of interest for all SLIDE-based analyses. Variance-stabilized counts obtained using the R package DESeq2 (86) were used as input for SLIDE analysis. Dataframes were created for each pairwise patient group comparison, resulting in 3 dataframes to separately undergo the following steps. To ensure the most highly variable features were input into SLIDE, only the top 2000 features for each dataframe were kept and input into SLIDE. Several models were fit to find the best combination of delta/lambda parameters. Performance of each model was judged both by model parsimony (i.e., fewer LFs) and a sample cross-validation performance. The use of permutation testing allows evaluation of model significance against a more rigorous dataset-specific null model, taking into account the correlation structure of the transcriptomic data. For all 3 comparisons, the optimal model had delta set to.075, and lambda set to 1. SPEC, a frequency-based parameter to quantify the stability of stages 1–2 of the multistage knockoff approach, was tuned for each comparison for performance. The SA-HC model used a SPEC of 0.3, whereas the SA-MMA and MMA-HC models used a SPEC of 0.15. The FDR was set to 0.1 and *F* (a feature split size) was set to the number of patients in any given comparison. Finally, after optimal models were chosen, each model was subjected to a full cross-validation, with 20 iterations and 4 folds.

The results of the full cross-validation confirmed that each of our models for the 3 pairwise comparisons were capable of distinguishing samples. The selection process described resulted in 3, 5, and 4 significant standalone LFs for the SA-HC, SA-MMA, and MMA-HC models, respectively. Based on rigorous permutation testing, interacting LFs did not add sufficient information to the classifier to justify their inclusion in the model, so models reported only included standalone LFs. Supplemental Figure 2 includes a list of features within each discovered significant LF.

### Cross-prediction in SARP.

The ability to predict SA-MMA labels in the SARP cohort was tested using the LFs discovered by SLIDE solely from the IMSA cohort for the SA-MMA comparison. This test is necessary to determine whether the LFs and performance observed within the IMSA cohort are generalizable. We generated a harmonized dataset including both SARP and IMSA participants (*N* = 160; SARP *n* = 128), which removed batch effects and standardized the data between cohorts prior to cross-prediction. However, for this harmonization, no labels were used at any point, ensuring no signal leakage. Only the top 2000 most variable features (identified in an unsupervised setting without any labels) after the original filtering (*n* = 2000) were retained. Features were not rescaled, ensuring the data remained harmonized. The SLIDE model for the SA-MMA discrimination task was trained solely using the IMSA cohort, and corresponding significant LFs were identified. Labels for the validation samples from SARP were then predicted using these significant LFs derived solely from the IMSA cohort. Both the structure and the weight of the LFs were identified based only on the IMSA cohort and they were not re-estimated for this cross prediction task ensuring no signal leakage. Performance of the IMSA-derived LFs in distinguishing between SA-MMA samples in the harmonized dataset indicated good generalizability, at AUC = 0.75 (95% CI: 0.66–0.81).

### Analysis of enrichment of biological pathways using GSEA and GSAI.

Correlation networks were used to visualize the structure of the discovered significant LFs. Threshold of edges shown are at an R of 0.4, and line width indicates magnitude of R. Nodes that are not connected to the network represent features which belong within the LF but do not have correlation strength above our threshold. In addition, features were passed through both GSEA using the R-package ReactomePA (87) and GSAI (54) to find Reactome pathways enriched within the LF sets for each model. For GSAI, all available LLMs supported by the web tool were utilized in search of pathways (GPT 4.0, CLAUDE, LLAMA, and MIXTRAL). Outputs needed to have at least medium-confidence, as assessed by the LLM to be included for consideration. Raw GSAI results were interpreted by a member of the research team for text to be created for a word cloud to summarize these results.

### Statistics.

All statistical analyses were performed in Stata/SE 18.5 (StataCorp). Graphs were produced in Prism 10.5.0 (GraphPad Software). For 2-way comparisons, normally distributed data were analyzed with a Student’s *t* test with Welch’s correction for variance; non-normally distributed data were analyzed with a nonparametric Mann-Whitney *U* test. Three-way comparisons were analyzed with the Kruskal-Wallis test with Dunn’s post hoc testing for between-group comparisons. Comparisons of categorical variables were performed with Fisher’s exact test. Comparisons of continuous variables were assessed for correlation with Spearman’s nonparametric *r*. Scatter plots show linear regression lines with 95% CIs shaded for visualization but reported statistical values are for Spearman’s *r*. Statistical significance was considered to be a *P* value of less than 0.05; however, exact *P* values are reported throughout.

### Study approval.

All research was approved by the University of Pittsburgh Institutional Review Board (IRB) study 19030100 and was performed in accordance with all relevant guidelines and regulations. Written informed consent was obtained from all participants prior to inclusion in both IMSA and SARP in accordance with the IRB at the University of Pittsburgh.

### Data availability.

All code and data are available at https://github.com/jishnu-lab/imsa_sarp_manuscript

Bulk RNA-seq data for airway brushings from the IMSA cohort have been deposited in the NCBI GEO (GSE158752). The corresponding authors may be contacted for the corresponding data for the SARP cohort. Values for all data points in graphs are reported in the Supporting Data Values file.

## Author contributions

AR and MCG conceived and designed the study and interpreted the data. SLK designed and performed experiments, analyzed and interpreted the data. JD supervised the design and execution of computational analyses and interpreted the data. AV and IM carried out all computational analyses. MAR designed, performed and processed imaging of lung tissue. MJC advised on quantitation of the imaging data. RPR helped to analyze data from the SARP cohort. HS helped with quantitation of imaging data. JCMP and HY helped with blinded analysis of imaging data. PGW and SAC performed RNA-sequencing and quality control on SARP epithelial brushings. MC, KS, SAC, NNJ, LCD, BG, ERB, DAM, WCM, EI, BDL, DM, and SE served as site PIs and supervised/performed bronchoscopies for the SARP network. AN helped with processing of human samples. TJN helped with human participant enrollment in study, collection of biospecimens and entry of patient metadata into our database. PR provided input into study design. CMSC provided oversight on tissue imaging. SEW provided critical review the manuscript. AR, MCG, JD, and SLK wrote the manuscript with input from co-authors.

## Conflict of interest

The authors report the following potential conflicts of interest: SLK, SEW, RR, MG, AR, AV and JD have a provisional patent application pending titled “METHOD TO BLOCK INCREASED EXPRESION OF MUCIN 1, STRESS KERATINS, AND LOSS OF LUNG FUNCTION IN SEVERE ASTHMA BY TARGETING GALNT5 AND/OR GALNT6.” MC has received grants from American Lung Association, Apogee Therapeutics, Apreo Health, AstraZeneca, Celldex, Connect BioPharma, Enveda, Expedition Therapeutics, Gala Therapeutics, Genentech, GlaxoSmithKline, Kinaset Therapeutics, Kinaset, Kymera, NIH, Nocion, Patient-Centered Outcomes Research Institute (PCORI), Pfizer, Pulmatrix, Sanofi-Aventis, Shionogi, Teva and Regeneron; has received consulting fees from Admin Physicians World, Allakos, Amgen, Apogee, Apreo Health, AstraZeneca, Blueprint Medicines, Citrus Health, Connect BioPharma, Enveda, Evommune, Genentech, Generate:Biomedicines, GlaxoSmithKline, Genzyme, Iqvia, ITA Group, Johnson & Johnson, Kymera, Meetings by Design, Novartis, Pfizer, Pioneering Medicines, Polarean, Regeneron, Sanofi-Aventis, Syneos, Teva, Uniquity Bio, Upstream Bio, and VIDA; has received honoraria for presentations from Amgen, AstraZeneca, Med Learning Group, Regeneron, and Sanofi-Aventis; and has stock in Aer Therapeutics. JD receives consulting fees from and has stock options in Seromyx. The following companies provided financial support for study activities at the Coordinating and Clinical Centers: Amgen, AstraZeneca, Boehringer-Ingelheim, Genentech, GlaxoSmithKline, Sanofi–Genzyme–Regeneron, and TEVA. These companies had no role in study design or data analysis, and the only restriction on the funds was that they be used to support the SARP initiative; as SARP site PIs, PGW, MC, KS, NJ, LCD, BG, ERB, DAM, WCM, EI, BDL, DM, SE and SEW benefited from this support.

## Funding support

This work was partially supported by the NIH and is subject to the NIH Public Access Policy of making the work publicly available in PubMed Central.

- NIH grants P01 AI106684 (to AR and SEW), R35 HL166219 and R01 AI176632 (to AR), K08 HL164887 (to MCG), R01 HL153058 (to SEW), and DP2AI164325 (to JD).
- NIH award number S10 OD028483 (to the High Throughput Computing cluster at the University of Pittsburgh Center for Research Computing and Data; RRID: SCR_022735).

## Supplementary Material

Supplemental data

Supplemental table 2

Supporting data values

## Figures and Tables

**Table 1:**
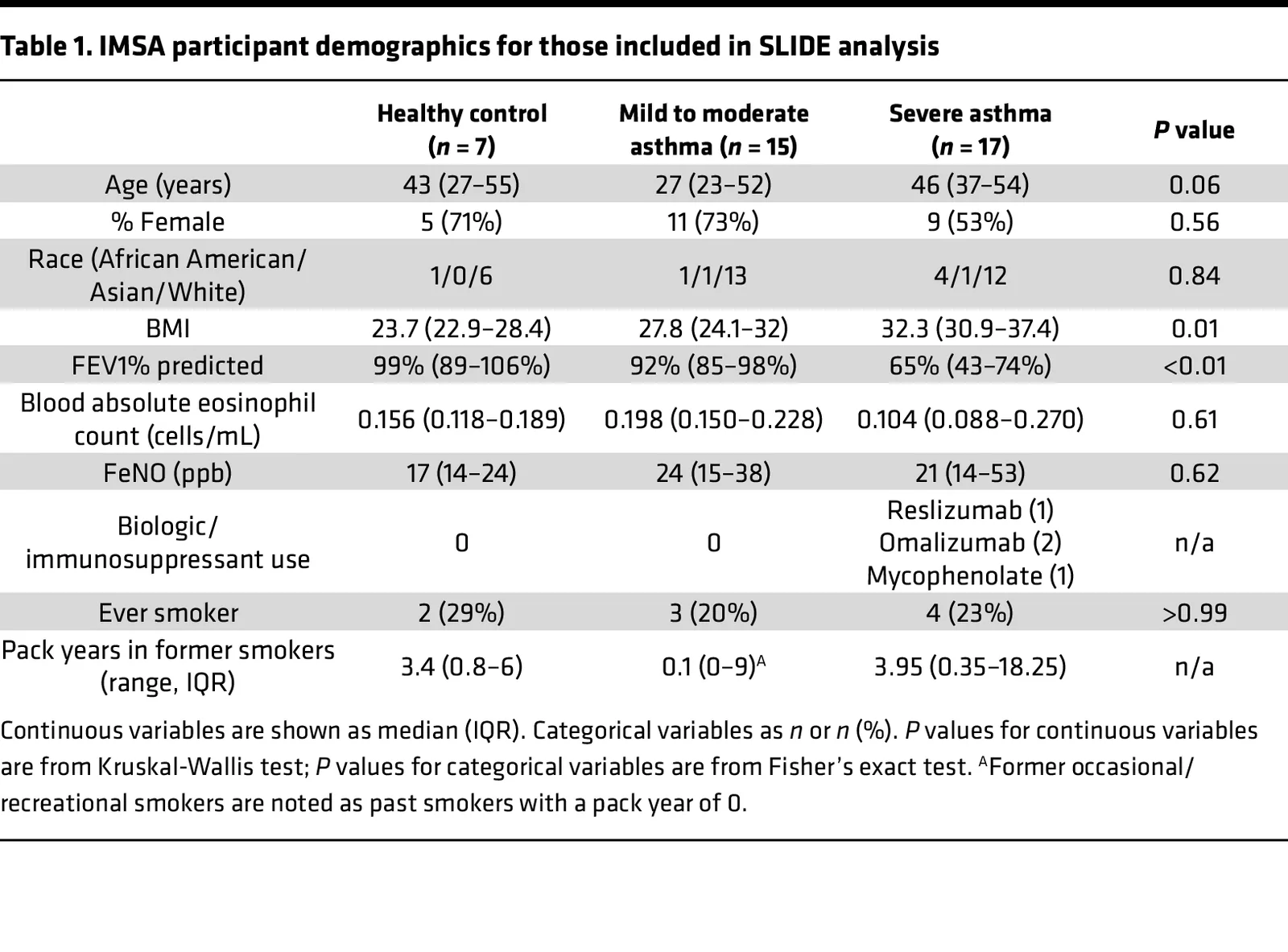
IMSA participant demographics for those included in SLIDE analysis

## Version 1 07/30/2026

In-Press Preview

## Version 2 08/24/2026

Electronic publication
