# Supplementary material for: Aberrant mucin expression and keratinization distinguishing severe from mild asthma revealed by interpretable machine learning
Source: JCI Insight. 2026 Jul 30;11(16):e202442. doi: 10.1172/jci.insight.202442 (PMC13502186; doi:10.1172/jci.insight.202442)
Supplement: Supplementary file 1 [file jciinsight-11-202442-s096.pdf]

Supplemental Table 1: SARP Participant Demographics (n=152)

		Healthy Controls (n=41, 27%)	Mild to Moderate Asthma (n=48, 32%)	Severe Asthma (n=63, 41%)	p-value
Age (years)		45.8 (29.1 – 52.8)	35.7 (27.4 – 47.1)	50.1 (35.9. 53.6)	0.01
% Female		24(59%)	33 (65%)	38 (63%)	0.83
Race	Native American	0 (0%)	1 (2%)	0 (0%)	0.19
	Asian	4 (10%)	1 (2%)	1 (2%)	
	African American	7 (17%)	13 (25%)	20 (33%)	
	White	29 (71%)	36 (71%)	37 (62%)	
	Hispanic	1 (2%)	0 (0%)	1 (2%)	
	Other	0 (0%)	0 (0%)	1 (2%)	
BMI		26.9 (23.3 – 30.0)	27.7 (22.8 – 33.6)	31.0 (26.1 – 37.8)	0.01
FEV1 % predicted		99.4% (90.6 – 109.9%)	88.1% (76.2 – 102.0%)	64.0% (56.0 – 81.0%)	<0.01
Blood Absolute Eosinophil Count (cells/mL)		130 (80 - 180)	176 (87 - 303)	175 (133 – 306)	0.04
FeNO (ppb)		15 (10 - 18)	31 (17 – 47)	20 (12 – 45)	<0.01

Continuous Variables are shown as median (IQR). Categorical Variables as n (%). P-values for categorical variables are Kruskal-Wallis; p-values for categorical variables are Fisher's Exact

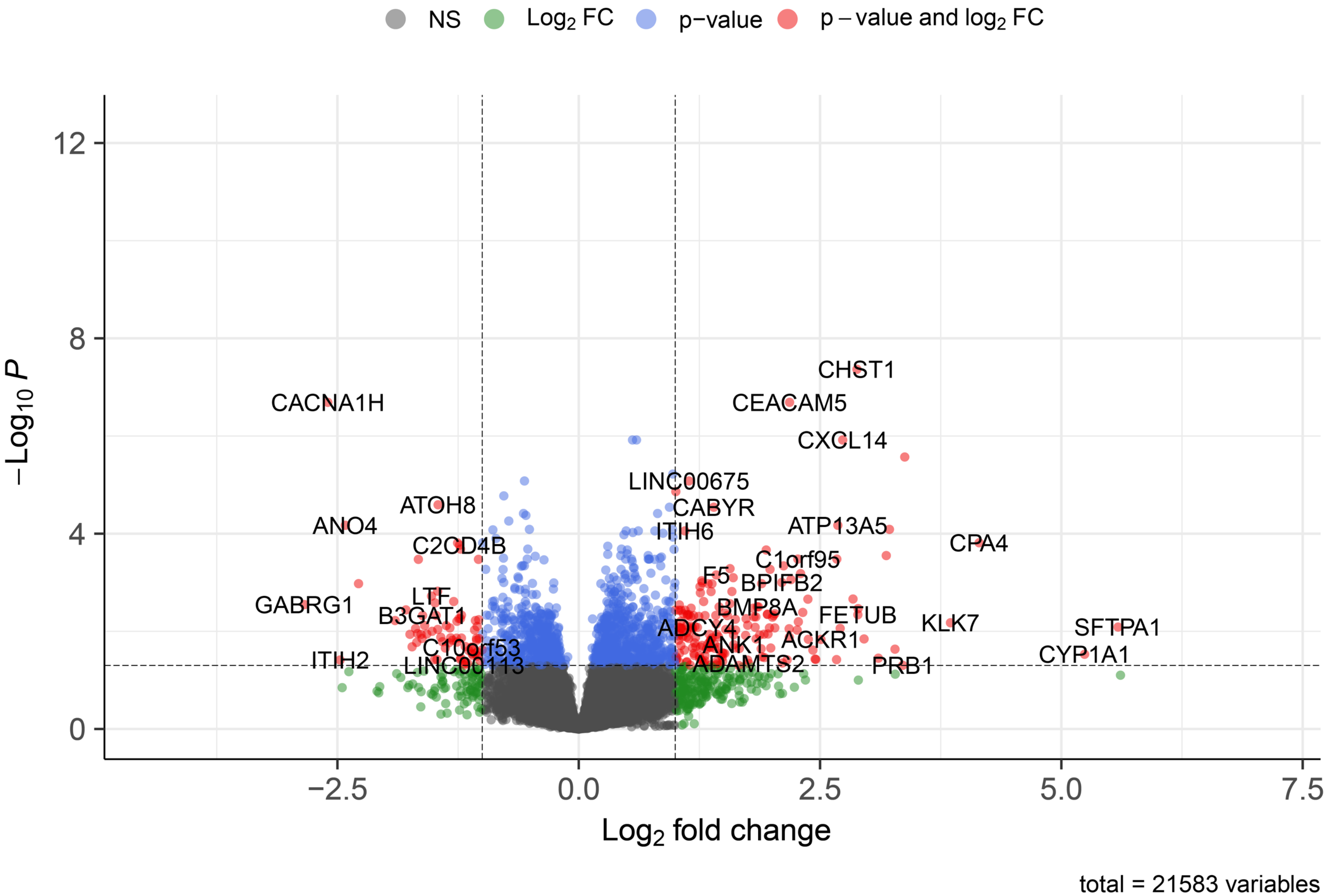

Supplemental Figure 1. Volcano plot showcasing differential expression results comparing MMA and SA using bulk RNA-seq data. Genes which are labeled or are represented by a red point have an absolute Log2 fold change greater than 1 and a p-adjusted value of < .05. Log2 fold change greater than 1 indicates greater expression in severe asthma patients. Plot generated using EnhancedVolcano package for R.

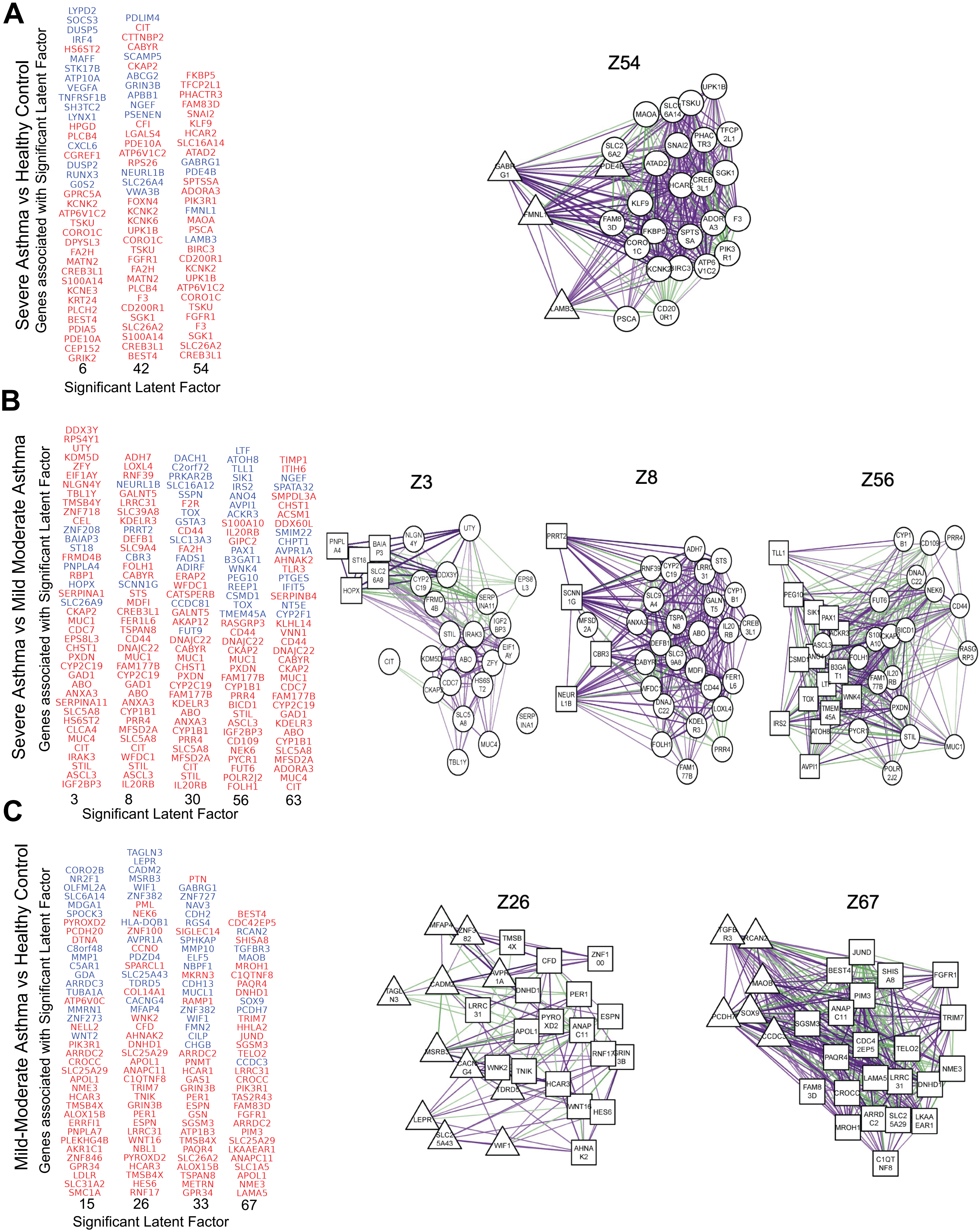
A
Severe Asthma vs Healthy Control
Genes associated with Significant Latent Factor
6: LYPD2 SOCS3 DUSP5 IRF4 HS6ST2 MAFF STK17B ATP10A VEGFA TNFRSF1B SH3TC2 LYNX1 HPGD PLCB4 CXCL6 CGREF1 DUSP2 RUNX3 G0S2 GPRC5A KCNK2 ATP6V1C2 TSKU CORO1C DPYSL3 FA2H MATN2 CREB3L1 S100A14 KCNE3 KRT24 PLCH2 BEST4 PDIA5 PDE10A CEP152 GRIK2
42: PDLIM4 CIT CTTNBP2 CABYR SCAMP5 CKAP2 ABCG2 GRIN3B APBB1 NGEF PSENEN CFI LGALS4 PDE10A ATP6V1C2 RPS26 NEURL1B SLC26A4 VWA3B FOXN4 KCNK2 KCNK6 UPK1B CORO1C TSKU FGFR1 FA2H MATN2 PLCB4 F3 CD200R1 SGK1 SLC26A2 S100A14 CREB3L1 BEST4
54: FKBP5 TFCP2L1 PHACTR3 FAM83D SNAI2 KLF9 HCAR2 SLC16A14 ATAD2 GABRG1 PDE4B SPTSSA ADORA3 PIK3R1 FMNL1 MAOA PSCA LAMB3 BIRC3 CD200R1 KCNK2 UPK1B ATP6V1C2 CORO1C TSKU FGFR1 F3 SGK1 SLC26A2 CREB3L1
Significant Latent Factor
Z54
B
Severe Asthma vs Mild Moderate Asthma
Genes associated with Significant Latent Factor
3: DDX3Y RPS4Y1 UTY KDM5D ZFY EIF1AY NLGN4Y TBL1Y TMSB4Y ZNF718 CEL ZNF208 BAIAP3 ST18 FRMD4B PNPLA4 RBP1 HOPX SERPINA1 SLC26A9 CKAP2 MUC1 CDC7 EPS8L3 CHST1 PXDN CYP2C19 GAD1 ABO ANXA3 SERPINA11 SLC5A8 HS6ST2 CLCA4 MUC4 CIT IRAK3 STIL ASCL3 IGF2BP3
8: ADH7 LOXL4 RNF39 NEURL1B GALNT5 LRRC31 SLC39A8 KDELR3 PRRT2 DEFB1 SLC9A4 CBR3 FOLH1 CABYR SCNN1G STS MDFI CREB3L1 FER1L6 TSPAN8 CD44 DNAJC22 MUC1 FAM177B CYP2C19 GAD1 ABO ANXA3 CYP1B1 PRR4 MFSD2A SLC5A8 CIT WFDC1 STIL ASCL3 IL20RB
30: DACH1 C2orf72 PRKAR2B SLC16A12 SSPN F2R TOX GSTA3 CD44 SLC13A3 FA2H FADS1 ADIRF ERAP2 CATSPERB WFDC1 CCDC81 GALNT5 AKAP12 FUT9 DNAJC22 CABYR MUC1 CHST1 PXDN CYP2C19 FAM177B KDELR3 ABO ANXA3 CYP1B1 PRR4 SLC5A8 MFSD2A CIT STIL IL20RB
56: LTF ATOH8 TLL1 SIK1 IRS2 ANO4 AVPI1 ACKR3 S100A10 IL20RB GIPC2 PAX1 B3GAT1 WNK4 PEG10 REEP1 CSMD1 TOX TMEM45A RASGRP3 CD44 DNAJC22 CKAP2 MUC1 PXDN FAM177B CYP1B1 PRR4 BICD1 STIL ASCL3 IGF2BP3 CD109 NEK6 PYCR1 FUT6 POLR2J2 FOLH1
63: TIMP1 ITIH6 NGEF SPATA32 SMPDL3A CHST1 ACSM1 DDX60L SMIM22 CHPT1 AVPR1A AHNAK2 TLR3 PTGES IFIT5 SERPINB4 NT5E CYP2F1 KLHL14 VNN1 CD44 DNAJC22 CABYR CKAP2 MUC1 CDC7 FAM177B CYP2C19 GAD1 ABO KDELR3 CYP1B1 SLC5A8 MFSD2A ADORA3 MUC4 CIT
Significant Latent Factor
Z3
Z8
Z56
C
Mild-Moderate Asthma vs Healthy Control
Genes associated with Significant Latent Factor
15: CORO2B NR2F1 OLFML2A SLC6A14 MDGA1 SPOCK3 PYROXD2 PCDH20 DTNA C8orf48 MMP1 C5AR1 GDA ARRDC3 TUBA1A ATP6V0C MMRN1 ZNF273 NELL2 WNT2 PIK3R1 ARRDC2 CROCC SLC25A29 APOL1 NME3 HCAR3 TMSB4X ALOX15B ERRFI1 PNPLA7 PLEKHG4B AKR1C1 ZNF846 GPR34 LDLR SLC31A2 SMC1A
26: TAGLN3 LEPR CADM2 MSRB3 WIF1 ZNF382 PML NEK6 HLA-DQB1 ZNF100 AVPR1A CCNO PDZD4 SPARCL1 SLC25A43 TDRD5 COL14A1 CACNG4 MFAP4 WNK2 CFD AHNAK2 DNHD1 SLC25A29 APOL1 ANAPC11 C1QTNF8 TRIM7 TNIK GRIN3B PER1 ESPN LRRC31 WNT16 NBL1 PYROXD2 HCAR3 TMSB4X HES6 RNF17
33: PTN GABRG1 ZNF727 NAV3 CDH2 RGS4 SIGLEC14 SPHKAP MMP10 ELF5 NBPF1 MKRN3 CDH13 MUCL1 RAMP1 ZNF382 WIF1 FMN2 CILP CHGB ARRDC2 PNMT HCAR1 GAS1 GRIN3B PER1 ESPN GSN SGSM3 ATP1B3 TMSB4X PAQR4 SLC26A2 ALOX15B TSPAN8 METRN GPR34
67: BEST4 CDC42EP5 RCAN2 SHISA8 TGFBR3 MAOB MROH1 C1QTNF8 PAQR4 DNHD1 SOX9 PCDH7 TRIM7 HHLA2 JUND SGSM3 TELO2 CCDC3 LRRC31 CROCC PIK3R1 TAS2R43 FAM83D FGFR1 ARRDC2 PIM3 SLC25A29 LKAAEAR1 ANAPC11 SLC1A5 APOL1 NME3 LAMA5
Significant Latent Factor
Z26
Z67

Supplemental Figure 2. Latent factor gene lists for each significant standalone latent factor as discovered by SLIDE and used in the analyses. Red features correspond to fold change in the direction of the higher severity for a given pairwise comparison, and vice versa for blue. To the right are correlation networks for latent factors not included in Figure 3 (labeled as Zx). Triangle nodes represent a logfc indicating greater expression in HC patients, whereas squares indicate greater expression in MMA and circles indicate the same for SA. Purple edges are positive correlation, and green edges are negative correlation. Edges are only present if they have a strength of R > |.4|. Edge thickness indicates strength of correlation. Nodes only present if they passed a loading and AUC threshold.

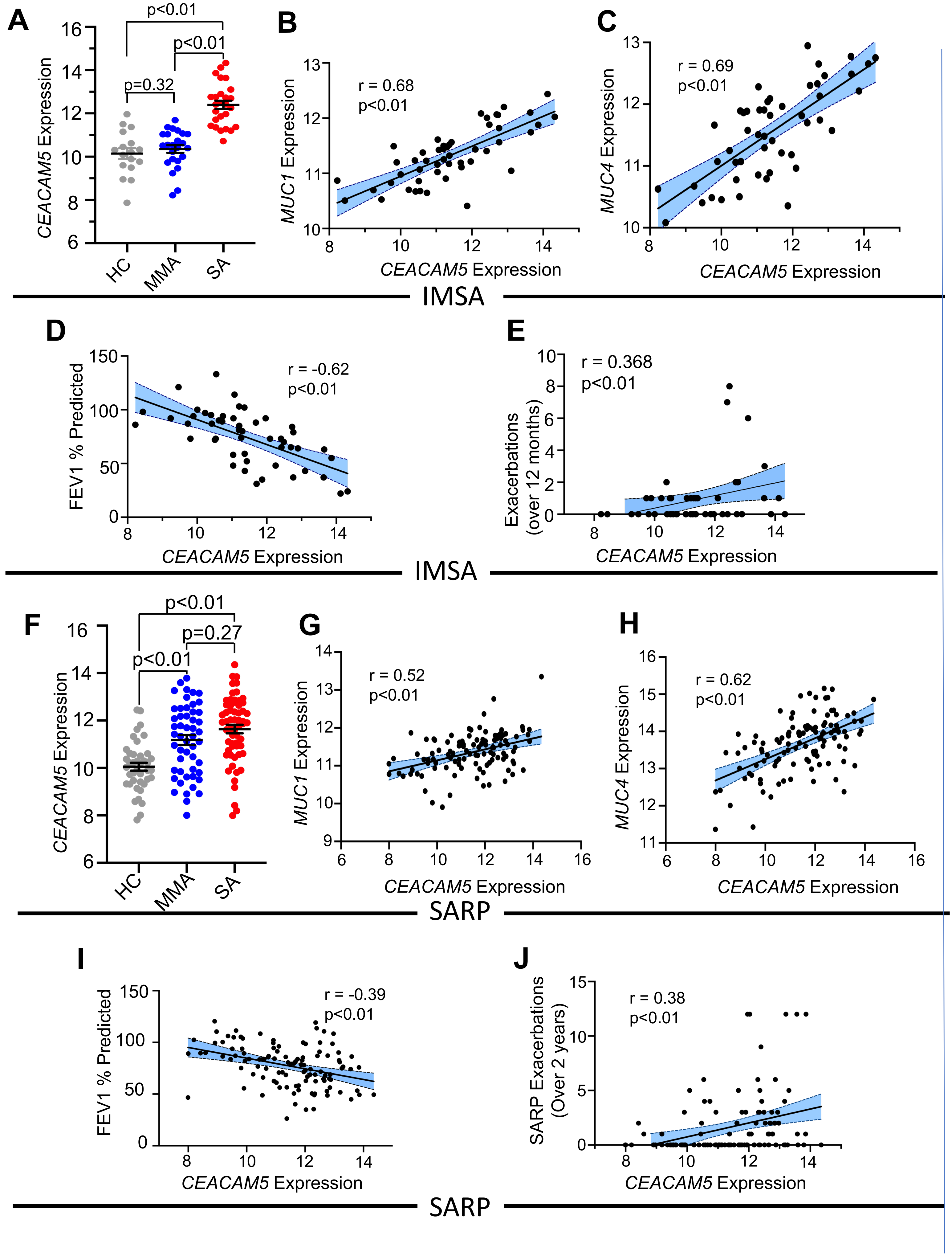
A
B
C
p<0.01
p<0.01
p=0.32
CEACAM5 Expression
HC
MMA
SA
r = 0.68
p<0.01
MUC1 Expression
CEACAM5 Expression
r = 0.69
p<0.01
MUC4 Expression
CEACAM5 Expression
IMSA
D
E
r = -0.62
p<0.01
FEV1 % Predicted
CEACAM5 Expression
r = 0.368
p<0.01
Exacerbations
(over 12 months)
CEACAM5 Expression
IMSA
F
G
H
p<0.01
p=0.27
p<0.01
CEACAM5 Expression
HC
MMA
SA
r = 0.52
p<0.01
MUC1 Expression
CEACAM5 Expression
r = 0.62
p<0.01
MUC4 Expression
CEACAM5 Expression
SARP
I
J
r = -0.39
p<0.01
FEV1 % Predicted
CEACAM5 Expression
r = 0.38
p<0.01
SARP Exacerbations
(Over 2 years)
CEACAM5 Expression
SARP

Supplemental Figure 3. Expression of *CEACAM5* gene and relationship with *MUC1* and *MUC4* gene expression and clinical measures in the two asthma cohorts. Shown are **(A)** *CEACAM5* RNA expression in bulk RNA-seq data of airway brushings by asthma severity and plotted against **(B)** *MUC1* and **(C)** *MUC4* expression**,** and asthma clinical correlations **(D)** FEV1% predicted **(E)** number of exacerbations in the 12-month period of cohort enrollment. **(F-I)** Similar data plotted using RNA-seq data of airway brushings in SARP. **(J)** Number of cumulative prospective exacerbations occurring from baseline visit through year 3 in SARP was plotted against *CEACAM5* expression. Data plotted as Mean + SEM. Spearman's nonparametric correlations, linear regression (solid line) with 95% CI (shaded blue) shown on each scatter plot. Kruskal-Wallis with Dunn's post-hoc testing, Spearman's nonparametric correlation.

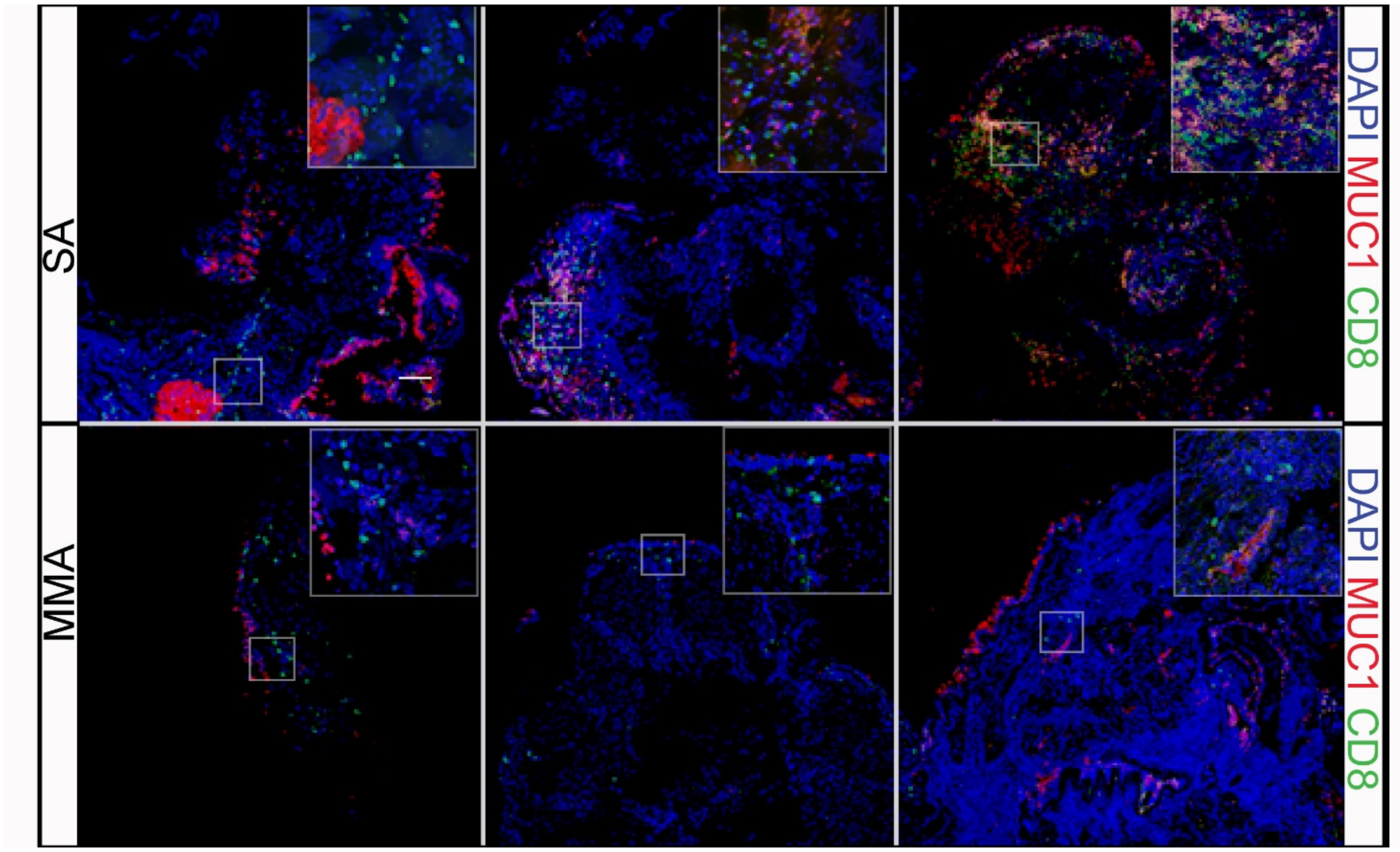

Supplemental Figure 4. Representative immunofluorescence images of MUC1 and CD8 proteins (inset). Images were derived from human endobronchial biopsies obtained from SA and MMA patients. Scale 200 μm.

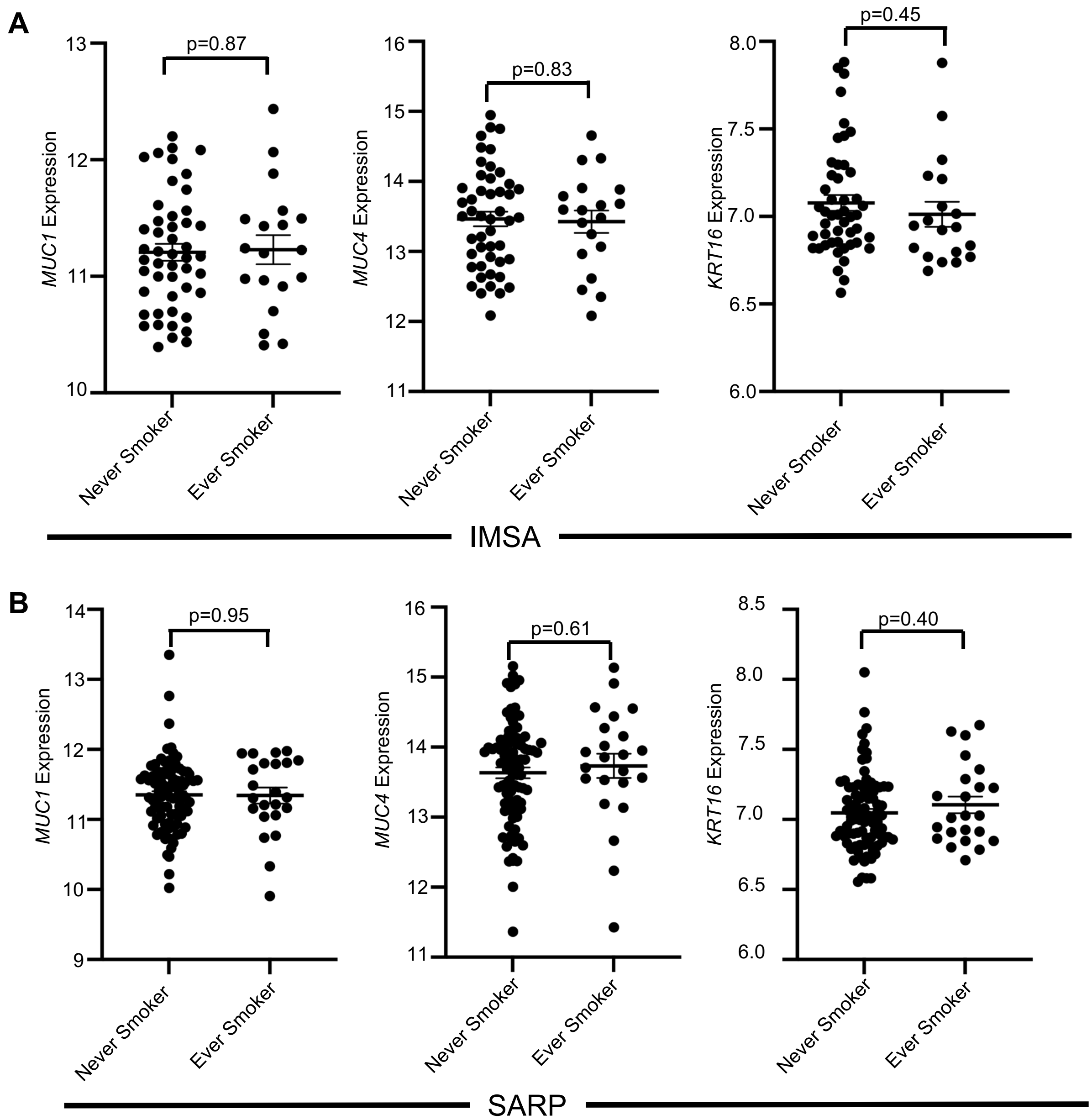

Supplemental Figure 5. Prior smoking history does not affect MUC1, MUC4 or KRT16 expression. Expression of MUC1, MUC4 and KRT16 compared in the IMSA and SARP cohorts between participants who reported never smoking (Never Smoker) and participants who had previously smoked but quit (Ever Smoker). Student's t-test with Welch's correction.
